# Animal Models in Eye Research: Focus on Corneal Pathologies

**DOI:** 10.3390/ijms242316661

**Published:** 2023-11-23

**Authors:** Alexis Loiseau, Gabrielle Raîche-Marcoux, Cloé Maranda, Nicolas Bertrand, Elodie Boisselier

**Affiliations:** 1 Faculty of Medicine, Department of Ophthalmology and Otolaryngology—Head and Neck Surgery, CHU de Québec Research Center, Université Laval, Québec, QC G1S 4L8, Canada; gabrielle.raiche-marcoux.1@ulaval.ca (G.R.-M.); cloe.maranda.1@ulaval.ca (C.M.); 2 Faculty of Pharmacy, CHU de Quebec Research Center, Université Laval, Québec, QC G1V 4G2, Canada; nicolas.bertrand@pha.ulaval.ca

**Keywords:** animal models, ocular research, cornea, corneal diseases, translational research

## Abstract

The eye is a complex sensory organ that enables visual perception of the world. The dysfunction of any of these tissues can impair vision. Conduction studies on laboratory animals are essential to ensure the safety of therapeutic products directly applied or injected into the eye to treat ocular diseases before eventually proceeding to clinical trials. Among these tissues, the cornea has unique homeostatic and regenerative mechanisms for maintaining transparency and refraction of external light, which are essential for vision. However, being the outermost tissue of the eye and directly exposed to the external environment, the cornea is particularly susceptible to injury and diseases. This review highlights the evidence for selecting appropriate animals to better understand and treat corneal diseases, which rank as the fifth leading cause of blindness worldwide. The development of reliable and human-relevant animal models is, therefore, a valuable research tool for understanding and translating fundamental mechanistic findings, as well as for assessing therapeutic potential in humans. First, this review emphasizes the unique characteristics of animal models used in ocular research. Subsequently, it discusses current animal models associated with human corneal pathologies, their utility in understanding ocular disease mechanisms, and their role as translational models for patients.

## 1. Animal Models in Ocular Research

In biomedical research, the establishment of animal models serves two key purposes: gaining an understanding of a pathological process and enabling the translational development of therapeutic or diagnostic modalities. Some exploratory models need to be characterized and adopted by the scientific community, in which researchers attempt to recapitulate a phenotype observed in humans in order to validate models and demonstrate the benefits of new therapies [1]. An appropriate animal model can elucidate some fundamental aspects of a human disease to promote a greater understanding of its mechanism. The model used does not need to replicate its human counterpart in every way but only in those aspects that are important for the advancement of knowledge. Like other domains of medical research, vision research focuses on the study of disease pathogenesis and the discovery of new therapies for the eye, using both in vitro and in vivo strategies. In vivo experiments involve animal models, including vertebrates (rodents, rabbits, primates, pigs, felines, canines, and zebrafish) and invertebrates (flies and nematodes) [2]. The development of relevant experimental models is, therefore, essential for identifying disease risk factors, elucidating the fundamental molecular mechanisms in disease progression, and providing guidance on the safety and efficacy of specific treatments.

**Figure 1:**
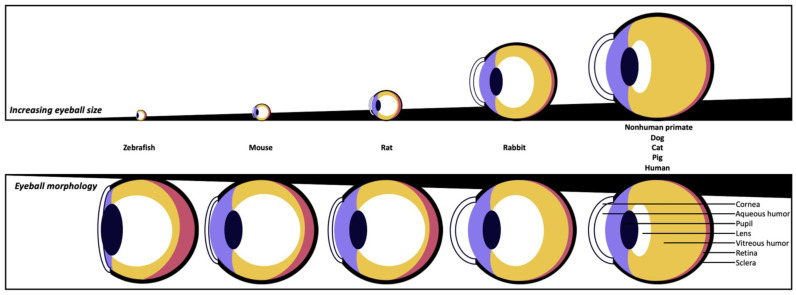
Relative comparison of eyeball size and morphology between different species used in eye research.

The eye is a complex system made up of numerous tissues integrated into a functional sensory organ that works together to make vision. The dysfunction of any one of these tissues can impair vision [3], arguably the most important of our senses. It is thus essential to conduct studies on laboratory animals to ensure the safety of therapeutics applied directly or injected into the eye to treat ocular diseases before eventually proceeding with clinical trials. Each animal has unique ocular characteristics that must be taken into account when extrapolating between species, even though many fundamental elements of the anatomy and physiology of the eye are highly conserved in vertebrates [1]. Differences in ocular anatomy between species can alter the way a drug interacts locally in the eye, whether administered directly into the eye (i.e., topically or intravitreally) or systemically [3]. Therefore, it is important to understand these differences and how they may influence the results and interpretation of safety or efficacy data for ocular therapeutics. Figure 1 and Table 1 compare some ocular anatomical parameters, especially in the anterior chamber, of different animals used in the laboratory. The eyes of nonhuman primates and humans are similar, although the nonhuman primate eye is a little more than half the size of the human eye in volume (approximately 4.0 cm^3^ vs. 7.2 cm^3^, respectively). The other species (rodents, rabbits, pigs, cats, dogs, and zebrafish) exhibit more noticeable differences in terms of lens size, vitreous body, anterior chamber containing aqueous humor, and corneal thickness [3]. Despite the description of various animal models used for common ocular diseases originating from the uvea, humor, lens, choroid, and retina (i.e., uveitis, glaucoma, cataract, age-related macular degeneration, diabetic retinopathy, and ocular tumors) [1,2,4,5,6,7,8,9], little information is available on corneal pathologies. However, corneal diseases are one of the most common causes of blindness worldwide [10,11,12,13]. Globally, an estimated 6 million people are blind and/or visually impaired because of corneal pathologies (e.g., infections, trauma, etc.) [10,13]. This review gathers the animal models most associated with the different corneal pathologies and offers relevant information to researchers for a judicious choice depending on the targeted eye condition.

**Figure 2:**
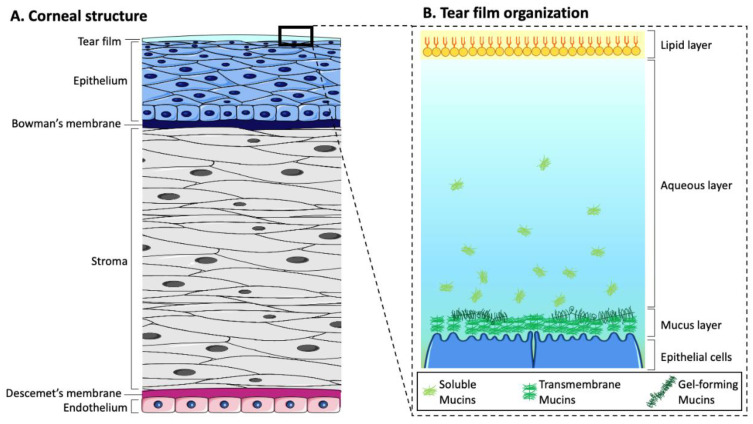
Structure of the human ocular surface. (**A**) The human cornea is composed of five distinct layers, three of which are cellular (epithelium, stroma, and endothelium) and two acellular (Bowman’s and Descemet’s membranes). (**B**) Tear film organization consists of a mucin-gel layer adjacent to the corneal epithelial surface, an aqueous layer containing mucins and other soluble proteins, and a thin lipid film on the outer surface.

The cornea is the outermost tissue of the eye and is exposed to the external environment. Thanks to its unique properties of transparency and avascularity, essential for vision, the cornea not only transmits light, it also refracts it, giving images sharpness [14]. Anatomically, the structure of the human cornea contains five main layers: the epithelium, Bowman’s layer, stroma, Descemet’s membrane, and endothelium (Figure 2). The species presented in this review have broadly the same corneal structure, although the proportion of each layer can be very different depending on the animal species [3,15]. Not all species have this Bowman’s membrane, such as rodents, rabbits, cats, and dogs (Table 1) [16,17].

**Table 1:** Relative comparison of selected ocular anatomical parameters of relevant laboratory animal species.

Species	Zebrafish	Mouse	Rat	Rabbit	Nonhuman Primate	Dog	Cat	Pig	Human
Average eye dimension in volume (cm^3^)	0.0035	0.025	0.1	2.6	4.0	4.5	5.1	6.5	7.2
Average eye dimension (axial length in mm)	2	3.4	6.0	17.1	19.8	20.5	21.4	23.9	24
Corneal horizontal diameter (µm)	2–2.5	3.1	5.1	13.4	10.2–11.4	13–17	15.5–18	14.5–16.5	11.8
Average central corneal thickness (µm)	16–20	90–130	160–200	350–400	420–460	500–600	545–650	600–1100	505–560
Cornea shape	Flat	Flat	Flat	Dome	Dome	Dome	Dome	Dome	Dome
Bowman’s membrane	Yes	No	No	No	Yes	No	No	Less developed or absent	Yes
Average tear volume (µL)	/	0.06–0.2	4.6	5–7.5	/	65	32	/	7–12
Average tear turnover rate (%/min)	/	5	/	6.5	/	12	11	/	15
Time between eye blinks	/	5 min	5 min	6 min	6 s	10–20 s	18 s	20–30 s	5 s
Nictitating membranes	No	Yes (non-functional)	Yes (non-functional)	Yes	No	Yes	Yes	Yes	No
Average aqueous humor volume (µL)	/	6	14	287	100–120	770	820–850	260–300	200–310
Lens size (axial length in mm)	1.0	2.2	3.8	6.4–7.9	3–4	6.7	7.7	7.4–7.8	4
Lens shape	Spheroid	Spheroid	Spheroid	≈Spheroid	Ellipsoid	≈Spheroid	≈Spheroid	≈Spheroid	Ellipsoid
Space taken by lens in eyeball	Very High	Very High	Very High	High	Low	Medium	Medium	Medium	Low
Fovea	No	No	No	No	Yes	No	No	Non-functional	Yes
References	[18,19,20,21,22,23]	[3,24,25,26,27]	[3,25,27,28,29]	[3,25,27,30,31,32,33]	[3,25,26,34,35]	[3,25,27,36,37,38,39,40,41]	[3,25,35,41,42,43,44]	[3,25,35,45,46,47,48]	[3,18,19,23,25,26,27,34,47,49,50]

Bowman’s membrane acts as a barrier protecting the stroma in modulating the passage of moderate- to large-sized proteins, preventing excessive swelling of the cornea, and has a mechanical role in maintaining corneal shape [16,17,51]. One of the specificities of the cornea is its natural transparency. Anything that interferes with the stromal architecture of the cornea can contribute to blood vessel migration, corneal pigmentation increase, corneal edema apparition, or disruption of the corneal transparency. The color, location, shape, and pattern of a corneal lesion can help determine the underlying cause of the disease. This review highlights the unique characteristics of animal models used in eye research before describing in detail current animal models associated with human corneal pathologies, their use to understand the ocular disease mechanisms, and as translational models to patients. It focuses on mouse and rabbit models, which constitute most of the models, along with more complex models designed for specific diseases and research strategies. The information summarized in Table 2 is described in detail in the following sections, taking two steps for each model. First, the similarities and differences with the human eye are outlined, and then their potential applications are discussed. The intention is to provide a valuable research tool for selecting reliable and relevant animal models for studying human corneal diseases.

### 1.1. Mouse Models

The mouse is the most commonly used mammalian model for research in terms of numbers due to their small size, favorable genetic manipulation, relatively short generation time, and affordable cost compared to other mammals [52,53]. Moreover, there are several ocular anatomical and physiological similarities between humans and mice, such as conventional outflow pathways [3,54], despite their evolution in different environments and differences in eye size (Figure 1 and Table 1) [55]. For example, the corneal and conjunctival epithelium have a stratified squamous structure in both cases, but the mouse corneal epithelium has an average of 13 cell layers, which is about twice that of humans; the epithelium represents 30% of the total corneal thickness in mice, compared to 10% in humans. It has also been shown that the mouse genome appears very similar to that of humans, not only in terms of the organization of genes on chromosomes but also at the level of individual genes and their DNA sequences, although their chromosome number and size differ from those of humans [55]. Thus, the impact of different molecules targeting certain molecular mechanisms may be slightly different between these two species [53,56]. Other advantages of the mouse model include its small size and weight, which significantly reduce the drug or chemical amounts needed for experiments. Mice can also breed in captivity with a high reproductive rate, have a short lifespan, can be easily held, and have a long history of cohabitation with humans. In addition, the low cost of purchasing and housing mice as laboratory animals, compared to other species, as well as the relatively low cost of inbred lines, also contribute to their frequent use in this field [52,57,58]. The average purchase cost is 60 CAD per animal, depending on strain, age, weight, source, and exposure to the research protocol, and the cost of per diem upkeep is approximately 1 CAD per cage in different institutions in Northeastern Canada (all per diems in this review reflect the reality in North America). It should be noted that costs can vary considerably when using transgenic mice and/or own breeding.

Globally, mice are predominantly used to understand biological processes and mechanisms, offering numerous possibilities to study the role of specific molecules, genes, proteins, and more [57]. Indeed, there are thousands of mouse models available to study different pathologies. They are widely used to study absorption, distribution, metabolism, excretion, and toxicity assays [59]. The mouse has become the most widely used model for studying the ocular surface and various pathological mechanisms such as epithelial repair [60,61], dry eye diseases (DED), also called keratoconjunctivitis sicca [62,63,64], as well as targeted therapeutic treatments [65]. Advanced immunogenetic research on the murine model as a prime model of the human immune system and the widespread availability of transgenic, mutant, or knockout strains (house mice that have been genetically modified to inactivate one or more genes in the embryonic stem cells from which they are derived) make mice a very attractive model for studying dry eye disease [66]. Further studies incorporating both intrinsic (immune, endocrine, neural, aging, sex) and extrinsic (environmental, contact lenses, exposure) factors in dry eye pathogenesis in mice represent a very significant advance in elucidating the fundamental mechanisms, as these many factors may contribute to the tear film hyperosmolarity and inflammatory cycle involved in dry eye [62,67,68,69]. Indeed, it is often difficult to attribute a single cause to most cases of dry eye, hence the importance of addressing all modifiable risk factors. However, the use of murine models can be questioned, in particular, because of the insufficient ability to mimic human conditions and transpose the observed results to humans [53,70]. In addition, the disadvantages of mouse models include the difficulty of studying corneal lesions through the lesser amounts of tissue harvested for testing because of their small eye size. Furthermore, the small volume of tear film in mice decreases the ability to detect molecules, proteins, and drugs, for example, even with the most sensitive techniques. This is particularly the case for the detection of biomarkers of infectious diseases such as herpes simplex virus type 1 (HSV-1) which is known for its ability to establish a permanent latent infection in humans after exposure to the virus.

### 1.2. Rabbit Models

Rabbit models have attracted more attention in recent years in biomedical and pharmaceutical research. The notion of size is a very important characteristic to consider when choosing the animal model used, and the rabbit is a convenient alternative model to large mammals [71,72]. Large animal models are notably easier to examine longitudinally when the animal is alive, allow more biological tissue to be collected in vivo or post mortem, and facilitate surgical procedures. Rabbits are therefore considered to be medium-sized laboratory animals, which requires the approval of a local animal ethics committee [71,72]. From a phylogenetic point of view, rabbits are closer to nonhuman primates than to rodents [73]. The value of rabbits as experimental models in biomedical research is likely to increase, as they have the potential to bridge the gap between rodents and large animal models, such as nonhuman primates (Figure 1). In addition, they share many similarities with humans, including a physiology with common cellular and molecular features, as well as a heterogeneous genetic background [74]. In general, rabbits are easy to handle and breed in the laboratory due to their gentle nature and represent one of the most cost-effective species among animal models [71,72]. In ophthalmology, rabbits share many anatomical features with humans, including eye size, internal structure, and optical system, as well as conjunctival cavity volume [3,71,75,76]. Among these characteristics, the size of rabbit eyes facilitates examination of the ocular surface to study the effect of medical and surgical procedures or to administer substances, for example [71,76]. It also makes the ocular surface more accessible for imaging and quantification by slip-lamp examination and facilitates the creation of corneal lesions comparable to those in humans. In addition, the large size of their eyes allows more ocular and neural tissue to be sampled, and their abundant tear film facilitates tear collection [76]. They may also exhibit decreased tear production and significant changes in the ocular surface, mimicking human eye diseases. Another important aspect is that, unlike rodents traditionally used for research, rabbits have a long lifespan, making it possible to study age-related eye diseases. In addition, the blood vessels of the rabbit retina are more superficial and, thus, more accessible for injections [3]. However, several unique and important ocular anatomical features must be considered when working with rabbit models. The anterior segment of the rabbit eye is larger, and its lens occupies a significant amount of space, leading to swelling of the iris and curvature of the anterior chamber (Figure 1) [3]. Rabbits are able to resist blinking over time due to the unique composition of their tears, produced by the Harderian gland, which is absent in primates [77]. The average purchase cost is 200 CAD per animal for rabbits weighing around 2 kg with unpigmented eyes (New Zealand Whites), which are the most used for eye research. The cost of per diem upkeep is approximately 3.50 CAD per animal in different institutions in Northeastern Canada. However, when compared to the mouse model, rabbit inbred strains are very expensive. The limitations to this approach with these models are thus more financial than scientific [57].

For all the above-mentioned ocular characteristics, the rabbit is an important model for morphological, mechanistic, and preclinical studies of common ocular diseases, including dry eye, glaucoma, age-related macular degeneration, retinopathies, and cataracts [71,78,79]. Additionally, the interesting lifespan of rabbits enables further study of the chronic symptoms involved in most of these eye diseases, linked to environmental and age-related factors, even if they remain shorter than the majority of clinical cases of the corresponding human eye diseases [71]. Rabbits are also especially useful for assessing the safety and efficacy of innovative approaches to the treatment of eye diseases, such as drug development, for example [72,78,79,80,81]. Finally, rabbit eyes are very well suited to performing various surgical manipulations, such as corneal transplantation, intraocular lens insertion, cataract extraction, or intravitreal and subretinal injection [71]. Research is therefore increasingly focused on the rapid development of rabbit genomics and proteomics, transgenic and knockout lines, as well as rabbit-specific reagents [72,82]. Few studies report proteomic analysis in rabbit eye research, such as the composition of the cornea [83,84] and tear film [85,86], a lens regeneration model to learn more about lens regeneration in humans after surgery [87], and proteome changes in aqueous humor following two types of cataract surgery incision procedures (clear corneal and limbal incisions) [88,89].

### 1.3. Nonhuman Primate Models

Nonhuman primates are the animal models that most closely resemble humans in terms of anatomy, physiology, genomic and proteomic composition, behavior, and life history due to their close phylogenetic affinity with humans [1,90,91,92,93]. Among the most widespread nonhuman primate species in research, macaques and baboons share over 90% of DNA and protein sequences with humans [94,95]. These two species also have important similarities in anatomy and physiology (Table 1), although baboons have highly advantageous anatomical features that more closely mimic those of humans, such as eye size, which is larger than that of macaques and most other monkeys [26]. In ophthalmology, the structure and organization of the visual pathways make nonhuman primates an ideal model for reproducing the human visual system and human eye diseases, particularly those affecting the retina [1,90,91,93,96]. Nonhuman primates have indeed been used to study accommodation and the effect of the visual environment on the development of eye refraction [97]. A large number of data have revealed that many behavioral adaptations are consequences of high retinal acuity [91]. Indeed, among mammals, only nonhuman primate models possess a macular with a fovea, an anatomical and functional specialization in the posterior segment of the retina shared with humans [91,96]. The organization of photoreceptor cells and the very high density of cone photoreceptors within the macular are unique features and are similar between humans and nonhuman primates, enabling high-resolution central vision [90,91,96]. Consequently, this animal model is undoubtedly the most suitable for understanding the complex process of vision and developing therapies to restore vision in humans, thanks to its functional anatomy of the visual system. However, experiments on nonhuman primates require special laboratory facilities, making them inaccessible to most researchers. The use of nonhuman primates is very costly, and the supply is limited due to ethical considerations in their care and the expenses involved in their purchase (an average of 5000 to 10,000 CAD per animal, depending upon age, weight, source, and exposure in multiple research protocols) [98,99].

Nonhuman primate models are widely used as highly relevant preclinical animal models to mimic human conditions, study pathogenesis, and develop treatments for complex ocular diseases such as diabetic retinopathy, choroidal neovascularization, wet age-macular degeneration, and glaucoma, or more specifically, for regulatory safety and toxicity studies [1,26,90,91,93,99,100,101]. Thus, the use of nonhuman primate models with their unique characteristics is a major step toward developing clinically effective therapies for patients. With the development of cell and gene therapy as well as genome-editing technologies, such as the CRISPR/Cas system, nonhuman primate models seem to be a highly appropriate model for reducing the time required to transpose these therapies into human clinical trials and considerably improving success rates [90,91,92]. Another important advantage of nonhuman primates for the development of therapies is the availability and similarity of ocular phenotyping instruments that enable assessment of the state of the anterior and posterior segment of the eye in living primates, notably with the use of optical coherence tomography [93]. These data can thus be compared and complement those obtained from human volunteers and patients.

### 1.4. Porcine Models

Anatomical and physiological similarities are observed in the size and structure of the eyeball between pig and human eyes, including the cornea and retina (Figure 1 and Table 1) [1,102]. As for the cornea, the main differences between pigs and humans lie in the absence of Bowman’s layer and the increase in central corneal thickness in pigs [1,47,102,103]. The pig eye has also been used in retinal studies due to the similarity of its retinal layers to those of the human retina and its holangiotic vascularization [1,104]. In addition, pig eyes have a visual band dominated by conical photoreceptors in the retina, with a density of these photoreceptors similar to that of humans in a region that anatomically mirrors the primate macula, although pigs have no true macula [47,104,105]. Disadvantages of the porcine model include the rapid growth of these animals, which makes long-term studies difficult. The orbit is small, making it difficult to surgically place extraocular devices [104]. It should also be noted that management, confinement issues, and price are the main reasons for the limited use of porcine models. The average purchase cost is 300 CAD per animal (depending on age, weight, source, and exposure to the research protocol), and the per diem rate is approximately 10 CAD per animal in different institutions in Northeastern Canada.

The pig’s eye is an ex vivo animal model often used in vision science research [103]. Ex vivo models offer excellent economic and logistical advantages over animal alternatives while providing valuable information without conducting costly and labor-intensive in vivo work. In particular, they enable rapid assessment of the safety and risks of chemical/pharmaceutical products [106]. In addition, the ex vivo porcine model remains the preferred model for surgical training of ophthalmology residents [107,108], and several corneal surgeries have been developed using ex vivo ocular tissues [109]. Although the pig is considered a large animal model, pig eyes are relatively easy to obtain from a butcher or slaughterhouse [102]. Moreover, there is a wide availability of low-cost porcine tissues [109]. As a result, the porcine model is particularly used in eye banking and tissue engineering studies, as well as in clinical, pharmaceutical, and toxicological research [109,110,111,112]. Nevertheless, certain considerations must be considered for ex vivo studies. The use of a post mortem porcine model often introduces “false positive” damage due to tissue degradation and preparation. In addition, the variation in central corneal thickness measured by ultrasonic pachymetry can be doubled depending on the age or type of pig slaughtered (Table 1) [1,47,102,103]. This difference could also be explained by corneal swelling if measurements are taken long after the animal has been slaughtered [47]. One of the limitations of using the pig model from the slaughterhouse or butcher is the impossibility of obtaining tears, which explains the lack of information on the subject [102]. Several investigations have also been carried out on the porcine model to study the anatomy, pathophysiology (corneal wound healing, dry eye, and stem cells), biomechanics, and immunology of the cornea [1,102,103,109,113]. Furthermore, the pig is the primary species used in xenotransplantation experiments involving cornea [1]. However, despite its widespread popularity, the porcine eye model is not used for full-thickness corneal transplantation contrary to feline model where corneal transplantation has been successfully performed (Section 1.5 and Section 2.3) [109,114,115]. Porcine corneas express galactose-α-1,3-galactose in anterior stromal keratocytes, an oligosaccharide which is not present in humans [1,113]. This is the main porcine antigen against which humans have performed antibodies that participate in graft rejection, mediated by the direct CD4^+^ T-cell response. Thus, there is a need to develop genetically modified porcine models to reduce the immunologic consequences of xenotransplantation and provide an unlimited supply of donor tissues [113]. In vitro studies suggest that models of porcine corneas lacking the α-1,3-galactosyltransferase gene, required for galactose-α-1,3-galactose expression, and also expressing one or more human complement-regulatory proteins (e.g., CD46, CD55 or CD59) are more protected against any humoral response during xenotransplantation (which would occur in the case of corneal neovascularization or inflammation) than wild-type corneas [113,116]. Moreover, porcine intestinal submucosa tissues are already used clinically as biomaterials as a collagen matrix to promote corneal remodeling and reconstruction of deep stromal defects in dogs, cats, and horses, but not in humans [1]. This model has also been used as cataract and glaucoma experimental model, as well as for retina and cataract surgeries [109].

### 1.5. Feline Models

Feline models, as large, long-lived animal models, are routinely used to test the safety and efficacy of emerging medical modalities, such as tissue transplantation from induced pluripotent stem cells [1,109,115,117]. The cat eye has many anatomical and physiological similarities with the human eye related to its overall structure and function (Figure 1 and Table 1) [118]. However, the feline eye is smaller than the human eye and has a much larger lens that occupies approximately 10% of the eyeball. Because the cat has a more spherical cornea, the cornea as well as the anterior chamber of the feline eye are also relatively larger than the same structures of the human eye [117,118]. The feline model, which is less available, more difficult to handle, and much more expensive than the mouse or rabbit models, is widely used in fundamental ophthalmology and neuroscience research due to similarities between the feline and human eye on retinal and visual function and structure, as well as on the entire optical system, including the visual tracts and the visual cortex [117]. The average purchase cost is 1000 CAD per animal (not including transportation costs corresponding to an additional 1000 CAD), depending on breed, age, weight, source, and exposure to the research protocol, and the per diem rate is approximately 10 CAD per animal in different institutions in Northeastern Canada.

The feline model has non-replicating endothelial cells similar to those of humans [115], and its use for the optimization of corneal transplantation techniques is well referenced since corneal transplantation has been successfully performed in feline models [109,114,115,119,120] (as described later in the Section 2.3). It is thus an interesting model for the preclinical assessment of corneal endothelial reconstitution by intracameral corneal endothelial cell injection [115]. The development of genomic resources in the cat has facilitated the mapping and further characterization of feline models for eye diseases, particularly at the molecular level [121,122]. With the recent increase in interest in purebred animals and the use of inbreeding in cat strains, inherited diseases affecting the eye have increased significantly in the feline population [121,122]. The inherited ophthalmic diseases of cats that have been characterized are lysosomal storage disorders, congenital glaucoma, and neuroretina degenerative diseases [117]. The cat is a promising resource of phenotypically defined genetic variations with significant biomedical significance. Feline models may, therefore, become the necessary last step in translational research for specific human ophthalmic diseases.

### 1.6. Canine Models

The similarity between dogs and humans is particularly relevant in the area of ocular pharmacology, with notable similarities in blink rate, tear turnover rate, and other factors relevant to drug delivery (e.g., globe volume, corneal thickness) (Table 1) [27]. However, differences must be considered in comparative studies, such as the presence of a nictitating membrane, larger tear volume, larger corneal size, and lower corneal elastic modulus in dogs. Moreover, dogs can be difficult to handle, which limits their applications in experimental ophthalmology. The most used dog breed for in vivo experiments is beagles or dogs of comparable size. The average purchase and maintenance costs per animal are close to those of the feline model, i.e., 1000 CAD (depending on breed, age, weight, source, and exposure to the research protocol; Section 1.5) in different institutions in Northeastern Canada.

Dogs are mainly used to study dry eye signs by measuring tear secretion [123]. Dog model presents decreased tear secretion and changes in the ocular surface, has longer lifespans than mice or rabbits, and offers better accessibility to its large ocular surface, easily accessible to clinical examination via the Schirmer’s test, which is the classic method for measuring tear production in humans [27,62,76,124]. The test can be performed using the same paper used for humans (Whatman no. 41 cellulose filter paper), but the test duration was limited to one minute to make it more convenient, whereas it is about 5 min in humans. These characteristics make the canine model extremely useful for pathophysiological studies as well as the development of therapeutic interventions for spontaneous keratoconjunctivitis sicca (also known as dry eye disease; DED). It is also possible to assess the stability of the tear film following dry eye pathologies, which is an interesting area of research, particularly in dogs or rabbits because of their similar corneal size to humans. The dog also appears to be a useful model for harvesting large quantities of mucus and conjunctival tissue to examine the ocular mucosal secretion system, which plays a vital role in ocular surface health by secreting lipids, water, mucins, growth factors, and antimicrobial peptides (from Meibomian and lacrimal glands, goblet, and epithelial cells) [76,125]. More recently, biosynthetic extracellular matrices have been implanted in dogs to determine the integration, innervation, and tolerance of these bio-interactive corneal matrices for future use in veterinary patients. In addition, they can serve as a model for the treatment of corneal diseases [126]. Indeed, non-healing erosions, vision-threatening corneal ulcers, and blinding corneal perforations in dogs are commonly seen in veterinary ophthalmology practices, and many of these diseases share several characteristics with their human counterparts [127]. Therefore, further clinical studies in dogs have the potential to benefit both human patients and veterinary practices.

### 1.7. Zebrafish Models

The zebrafish represents a valuable model organism for studying human ocular development and some eye diseases [18,19,128,129,130]. Zebrafish allows combining advantages of invertebrate models with those inherent to vertebrates for modeling human physiology. It has a short generation time of 2–4 months, can easily reproduce with a very high reproductive rate (200 offspring on a weekly basis), can be maintained in a relatively small space, has high homology with human genes (70% of human genes have at least one obvious zebrafish ortholog, compared to 80% of human genes with mouse orthologs) and has a low relative cost of raising zebrafish, making it an ideal model organism. Moreover, the average cost per year is about three times less for zebrafish [131]. The average purchase cost is 2 CAD per animal, and the cost of the facility varies from about 250 to 1000 CAD, depending on the size of the facility and the experiments that will be conducted [132], with a daily rate of about 1 CAD per tank in different institutions in Northeastern Canada. In the context of ophthalmological research, zebrafish eyes are like human eyes in terms of morphology, physiology, gene expression, and function (Table 1) [18,128,130,133,134]. Indeed, the analysis of the embryonic development of the eye posterior segment [128], which includes the neural retina and the retinal pigment epithelium as well as the eye anterior segment [128,135,136,137] (which includes the lens, cornea, ciliary body), highlights the sequence of events in vertebrate eye development but also similarities in the architecture between zebrafish and human eyes. However, the zebrafish retina retains its ability to regenerate throughout life, unlike mammals [1].

The zebrafish is mainly used to understand underlying developmental processes, to identify potential causative genes for human disorders, and to develop new therapies in the field of ophthalmic medicine [19]. Zebrafish has proven to be a powerful tool for the genetic analysis of visual system development and function, making it an attractive alternative to model species such as mouse for the study of ocular genetics [129,133,134,138]. This model is especially used as a model for human ocular diseases such as glaucoma, cataracts, photoreceptor degeneration, corneal dystrophies, and retinal pigmented epithelium [18,19,128,129,130]. In addition, zebrafish have become an attractive model for ocular preclinical drug toxicity testing and are now increasingly used for the discovery of novel treatment approaches [18,19]. This animal model has already been described to predict drug-related oculotoxicity at the preclinical stage, which is crucial because it can occur with the use of systemic, intravitreal, or topical drugs [139]. Due to the many advantages inherent to this model organism, zebrafish is also highly suitable for high-throughput screening approaches to identify novel avenues in the field of ophthalmic medicine [19].

## 2. Focus on the Most Used Animal Models in Corneal Pathologies

Corneal diseases are the fifth leading cause of blindness worldwide, after cataracts, refractive errors, glaucoma, and age-related macular degeneration [12]. Thus, this section brings together the different corneal pathologies with the most relevant and widely used animal models. The main objective is to highlight the evidence for the choice of animals to better understand and cure these corneal diseases. Table 2, which gathers information described in the following sections, summarizes the different animal models most used to study, understand, and benefit the patient from translational research in corneal diseases.

### 2.1. Dry Eye Diseases

#### 2.1.1. Pathology

**Figure 3:**
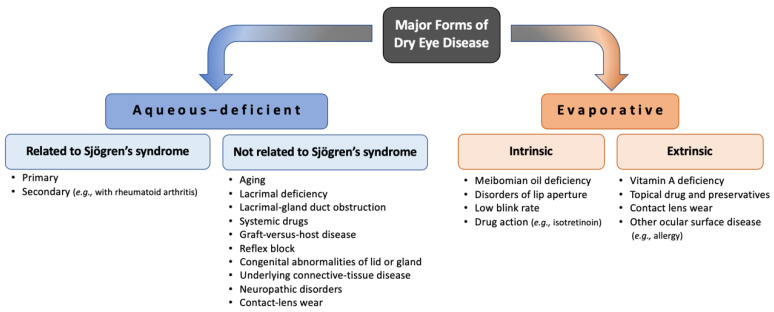
The two main enteropathogenic classifications of dry eye.

Dry eye disease (DED) is the most frequent disorder in ophthalmology. DED is a multifactorial tear film and ocular surface disease causing both objective and subjective symptoms [140,141], mainly due to insufficient tear production, excessive tear evaporation, or goblet cell loss [142,143]. Destabilization of the ocular surface mucosa, composed of mucins, is one of the main causes of dry eye [55,59,144]. Mucins are secreted by conjunctival goblet cells and lacrimal glands and are expressed at the membrane level of corneal and conjunctival epithelial cells. They are essential for maintaining the wettability of the ocular surface and thus contribute to the dynamics, stability, and osmolarity of the tear film. Thus, alterations in mucin expression can lead to increased water loss from tears, ultimately contributing to tear hyperosmolarity, which has been associated with ocular surface inflammation [59,144]. The glands involved in the tear film secretion are the lacrimal glands, which produce and remove the aqueous layer, while the Meibomian glands produce the lipid layer (Figure 2). This lipid layer is then spread over the tear film with each blink to stabilize it [59]. Therefore, tear film secretions from the lacrimal and the Meibomian gland contribute to the mucin content of the ocular surface. On the one hand, objective signs are tear film instability with the potential for ocular surface damage, increased osmolarity of the tear film, mucus discharge, increased frequency of blinking-tearing, and ocular surface inflammation [140,141,143]. On the other hand, subjective symptoms include visual disturbances (i.e., blurred) and discomfort (sensations of dryness or foreign body, pain, irritation, redness, burning, itching, sensitivity to light, and intolerance to contact lenses) [140,141,145]. Unlike objective signs, which are quantifiable and can also be assessed in animal models, subjective symptoms are evaluated using questionnaires and visual function tests (visual acuity at high and low contrast, dynamic visual acuity, and contrast sensitivity) in the patient [62,76,140,141,143,145,146]. Numerous studies have shown that psychological effects (depression, anxiety, feelings of happiness, etc.), personality traits, and the patient’s sensitivity to pain influence subjective symptoms of DED [140], making it difficult to use and interpret these symptoms in animal models. As an example, there is evidence of a relationship between patients’ subjective happiness and reported dry eye symptoms [147]. One study showed that a more enriched environment (e.g., exercise, well-being, sensory, cognitive, and social stimuli) in a stress-induced DED mouse model compared to a standard environment provides an effective intervention to prevent and attenuate decreased tear secretion in DED [148]. Behavioral observations can also be used to assess visual acuity in DED animal models using rodents or nonhuman primates [91,149]. Moreover, several factors can affect the DED severity, including autoimmune diseases and hormonal changes that play an important role in regulating tear production by the lacrimal gland [150], environmental surroundings (pollution), daily activities (watching TV, reading, mobile devices or computer), contact lens use, anatomical features, chronic inflammation, infections, and iatrogenic factors, such as medications or surgery [143,151,152]. The global prevalence of DED is from 5% to 50% with a higher rate in women than in men [153]. In 2017, the report from the Tear Film and Ocular Surface Society International Dry Eye Workshop II defined and updated the classification of DED into two main classes: aqueous-deficient dry eye and evaporative dry eye [154]. Although aqueous-deficient dry eye and evaporative dry eye show similar signs of reduced stability and increased osmolarity of the tear film, aqueous-deficient dry eye refers primarily to a failure of lacrimal secretion and evaporative dry eye is due to excessive water loss from the exposed ocular surface in the presence of normal tear secretory function [155]. It is important for clinicians and researchers to consider both forms of dry eye when diagnosing, treating, monitoring, and establishing animal models of DED because risk factors, causes, and treatments vary according to forms and subtypes (Figure 3). Drugs used to treat this disease account for around 15% of the global ophthalmic pharmaceutical market [145]. In addition, the annual cost of dry eye management, including direct healthcare costs (medication and doctor visits), impact on patient quality of life, and reduced work productivity, is estimated at 3.84 billion USD in the US alone [156].

#### 2.1.2. Animal Models

**Figure 4:**
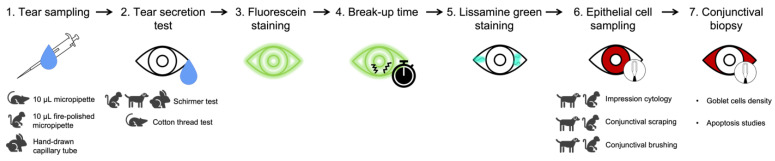
Suggested steps to assess tear film and ocular surface in animal models of dry eye.

Several animal models of dry eye have been established to mimic the different characteristics of these diseases, inducing the clinical manifestations by different pathways (mechanical or surgical approaches, neural pathway blockage, topical eye drops, iatrogenic immune response, desiccating stress, etc.) [157]. In the case of desiccation stress, animals are housed in a controlled environment where humidity, temperature, and airflow are regulated (from a few weeks to several months) to disrupt the immune homeostasis of the ocular surface to induce ocular dryness [65,158,159]. The most common animals used to establish DED models are mice, rats, and rabbits, whereas the use of dogs and primates is less frequent [66,160]. On the one hand, mouse models are extremely attractive models for this type of pathology (low cost and various gene knockout models). However, the anatomical and physiological features of mice still constitute a challenge for ocular tissue dissection and DED drug development, as ocular drug biodistribution studies are less representative of human reality (Figure 1 and Table 1) [157]. An important link between drug delivery and the mucin content study was observed, as the corneal and conjunctival epithelium are the primary absorption tissues for these drugs. Membrane-associated mucins may decrease or increase ocular bioavailability depending on the extent of their role as barrier or retention sites. Ocular barrier function in mice is equivalent to that in humans, despite the substitution of MUC16 (MUC for mucins in human) by Muc4 (Muc for mucins in mouse) in mouse corneal epithelial cells and the fact that extracellular domains of Muc4 and Muc16 are shorter than those of MUC4 and MUC16, respectively [55,65]. Therefore, the mouse model can provide an interesting model for studying factors on drug pharmacokinetics. On the other hand, the histoarchitectural features of the rabbit lacrimal gland more closely resemble those of the human lacrimal gland compared to murine models. Rabbit eyes also allow better accessibility to the ocular surface, thus facilitating phenotypic observation [160]. Indeed, clinical assessment, commonly used in humans to diagnose and evaluate objective symptoms of DED, has been particularly used in rabbits and dogs because of their large exposed ocular surface [27,76,161]. There are few methods available to assess the objective signs of dry eye in humans or animals, such as the Schirmer’s test (measuring the aqueous tear production in a given time), tear break-up time (demonstrating tear instability) and fluorescein, lissamine green, or rose Bengal staining (demonstrating ocular surface damage) [27,76,161]. Nevertheless, standardized procedures for these assessments have not been established for testing new therapies and full diagnosis of dry eye in ophthalmic research using DED animal models. For example, the size of the mouse eyeball does not allow the use of these tests without prior modification [76,143]. Additional techniques are also used to further study the ocular surface for clinical diagnosis of dry eye, such as the characterization of the tear film osmolarity, the chemical composition of the tear film, the mucins expression, or the ocular surface inflammation by demonstrating higher expression of conjunctival apoptotic markers [76]. A possible sequence of tests was suggested by Barabino et al. based on commonly used clinical tests and animal models to fully characterize dry eye after highlighting their intrinsic advantages and limitations (Figure 4) [76]. However, an attempt should be made to translate each clinical test to each animal species to optimize the use of animal models and take advantage of each species in dry eye studies. Certain models are suitable for studying DED pathogenesis, whereas other models are more appropriate for examining the therapeutic effects.

##### Mouse Models

**Figure 5:**
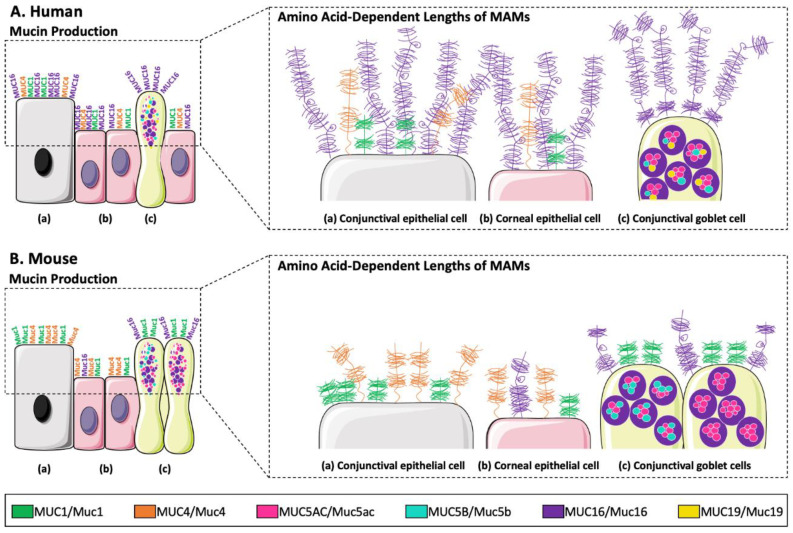
Major differences in mucin production by (**a**) conjunctival epithelial cells, (**b**) corneal epithelial cells, and (**c**) conjunctival goblet cells, and in amino acid-dependent lengths of membrane-associated mucins (MUC1/Muc1, MUC4/Muc4, and MUC16/Muc16) between (**A**) the human and (**B**) the mouse ocular surface.

The production of mucins in mouse models and their role on the ocular surface have been recently highlighted [59]. Portal et al. reviewed the different mucin expressions using DED mouse models [59]. These mucins, conferring the rheological properties of the mucus gel, are similar between humans (MUC) and mice (Muc) [59,162]. The three main membrane-associated mucins of the human ocular surface are MUC1, MUC4, and MUC16 (Muc1, Muc4, and Muc16 for mouse). In humans, MUC1, MUC4, and MUC16 are produced in both the cornea and conjunctiva [163,164] as well as in the mouse [165,166,167], with the exception of Muc16 which is produced only in the conjunctiva [168]. Human lacrimal glands produce mucins MUC1 (also produced by the Meibomian glands), MUC5AC, MUC5B, MUC7, and MUC19, whereas in the mouse, Muc1, Muc2, Muc3, Muc4, Muc5ac, Muc5b, Muc6, Muc10, Muc13, Muc14, Muc15, Muc16, Muc19, and Muc20 are expressed in Meibomian glands [59]. Until now, mucin production by ocular glands in mice has not yet been studied/analyzed at the protein level. The main differences in mucin production between humans and mice are presented in Figure 5.

One of the most notable differences between humans and mice is the difference in length of membrane-associated mucins in terms of amino acid number (which are shorter in mice with factors of 0.6, 0.5, and 0.5 for Muc16/MUC16, Muc4/MUC4, and Muc3/MUC3, respectively) as shown in Figure 5. The mucin that appears to be the most different between the two species is Muc16/MUC16 [55]. Indeed, MUC16 is larger and evenly distributed around the cornea in humans compared to Muc16 in mice. Furthermore, transgenic mice provide an important complement to cell culture models on the biological role of membrane-associated mucins. Knockout mice were genetically engineered not to overexpress membrane-associated mucins such as Muc1, Muc4, Muc13, and Muc16. Deficiency of Muc1 and Muc16 membrane-associated mucins can cause inflammation of the gastrointestinal tract but also of the ocular surface [55,169]. The variety of transgenic mice allows us to learn more about the molecular mechanisms of diseases [66,69].

As described previously, aqueous-deficient dry eye and evaporative dry eye are the main forms of dry eye. For aqueous-deficient dry eye, surgical models have been developed, such as excision of the extra orbital lacrimal gland [157,170] and/or cauterization of the lacrimal duct [171] as well as physical models by placing mice in a desiccating environment with or without transdermal application of scopolamine [63,157], or by subjecting mice to a therapeutic dose of radiation [172]. Chemical models also exist through topical applications or injections of drugs to mimic dry eye [59,157,173]. Among these drugs, benzalkonium chloride is one of the most commonly used to induce DED by topical application and cause goblet cell loss, associated with increased corneal thickness, apoptosis, corneal inflammation, and neovascularization [59,157,174,175]. Other drugs are used such as botulinum toxins ((BTX)-A and (BTX)-B), which are neurotoxins that can induce a localized clinical condition of dry eye without systemic side effects when periorbitally injected [59,176], or more recently, *N*-acetylcysteine, which is a mucolytic agent capable of inducing ocular surface damage and tear film instability by prompting MUC16 disruption and release from the ocular surface [157,173]. Chemical methods have the advantage of using a smaller number of animals since they allow one eye to be used as a dry eye model and the other as an internal control. Many genetic mouse models have also been reported for aqueous-deficient dry eye models, including *Sod1^−^/^−^* and *NHE8^−^/^−^* (decreased goblet cell density and Muc5ac expression), *NRTN^−^/^−^* (decreased goblet cell density and expression of Muc1 and muc4), *Klf4CN* (goblet cell absence), *Spdef^−^/^−^* (decreased expression of Muc5ac and Muc5b), and *Tet-mev-1* (induction of excessive oxidative stress associated with damage to ocular surface epithelium and reduced aqueous secretion function) [59]. With regards to evaporative dry eye, the models used are mainly genetically modified mice that have no or abnormal Meibomian glands [59]. In addition, a mouse model has been proposed to study the level of homeostatic protein clusterin (expressed in both human and mouse corneal epithelial cell layers and in the human tear film). Clusterin is probably responsible for inflammation, which induces severe dry eye when its level decreases below the critical threshold (between 3 and 6 μg/mL in a preclinical mouse model using desiccating stress) [55,65].

##### Rabbit Models

Various rabbit models have been developed to study DED. A rabbit model was established to simulate Sjögren’s syndrome, a chronic and multisystemic autoimmune disease characterized by lymphocytic infiltration of the exocrine lacrimal glands, leading to the classic manifestations of dry eye (Figure 3) [157,161]. To this end, autoimmune disease can be induced by co-cultivating autologous lacrimal gland cells and peripheral lymphocytes in vitro and injecting them into the lacrimal glands. This procedure allows their dysfunction and induces the symptoms of DED in this model. Other studies have used “short-term” rabbit models for evaporative dry eye by mechanically preventing rabbits from blinking using eyelid specula or stitches [62,157]. Due to the use of anesthetics, which may decrease tear secretion, and the induced dry eye acuity, this model is not optimal for studying the pathogenesis of DED, which is a chronic event. However, this model has the advantage of easily and efficiently generating the clinical symptoms of DED in just two hours. Thus, it is very useful for screening and comparing topical eye drops that help maintain cornea hydration, such as artificial tears or other therapies aimed at delaying evaporative loss of the preocular tear film [124]. Moreover, surgical approaches have also been used to develop different DED rabbit models, such as lacrimal or Meibomian gland dysfunction models [177,178,179,180]. Surgical closure of the lacrimal gland excretory ducts or Meibomian gland orifices by cauterization can increase tear evaporation or decrease tear secretion, reflecting a higher electrolyte concentration on the ocular surface. An increase in tear osmolarity on the first postoperative day, accompanied by a significant decrease in conjunctival goblet cell density after 8 weeks, was observed after closure of the lacrimal gland excretory ducts and surgical removal of the nictitating membrane and Harderian gland in rabbits [76]. Moreover, it has been shown that the closure of Meibomian gland orifices is a feature of Meibomian-related dry eye, as seen clinically in several ocular diseases [180]. More recently, a rabbit model in which dry eye is induced by mitogen concanavalin A injection into the orbital lacrimal glands of rabbits has been established [78,79]. Concanavalin A induces a strong inflammatory response and destruction of the lacrimal gland structure, creating a clinically relevant situation of acute DED. On the third day after the concanavalin A injection, results showed that tearing was reduced by around half. This model also showed that after a single injection of concanavalin A, induced DED lasts for around a week. However, it is possible to make this model chronic (from days to weeks) for DED by injecting concanavalin A weekly to prolong dry eye symptoms, which are reproducible and consistent for at least 3 weeks. Standard clinical tests for dry eye such as tear break-up time and fluorescein or rose Bengal staining of the ocular surface can be performed much more easily in rabbits due to the large exposed ocular surface and globe size compared to small animals such as mice and rats (Figure 1 and Table 1) [62,76,161]. To have a high chance of acceptable reproducibility under the conditions of Schirmer’s test in rabbits, Barabino et al. suggest a 1-min test without the use of anesthetics [76]. Indeed, it is difficult to compare different conditions used in Schirmer’s test due to the high variability of parameters. For example, the test duration, the use of anesthesia or not, the ease with which the animals are handled, and the opening and blinking of the eyelids can vary the results obtained via Schirmer’s test. Instead of using usual methods such as fluorescein or rose Bengal staining, Goto et al. developed a new tear film stability analysis system using videokeratography [181]. This noninvasive and objective tear break-up time method showed a better sensitivity for tear film stability analysis by capturing consecutive corneal surface images every second, and this technique represents an interesting research area with rabbit models of dry eye due to their cornea size [76]. However, the development of adapted software is required for the specific corneal curvatures of this animal. Therefore, the rabbit model is suitable for the study of lacrimal physiology and pathophysiology of DED, as well as for the efficacity and safety evaluation of therapeutic agents.

### 2.2. Ocular Herpes (Herpetic Keratitis)

#### 2.2.1. Pathology

Herpes simplex virus (HSV) is a widespread viral pathogen that infects most of the world’s population. This contagious infection is transmitted by simple contact with a person carrying the virus or by self-contamination. HSV is a ubiquitous human pathogen represented by two distinct serotypes—HSV-1 and HSV-2—which account for 90% and 20–25% of adult seropositivity, respectively [182]. Many primary infections are asymptomatic, making HSV infections in humans difficult to detect and study. Although oral and genital lesions are the most common manifestations of infection, HSV-1 can also affect ocular tissues, including the cornea, eyelids, conjunctiva, uveal tract, and retina [183]. HSV-1 ocular infection is the leading infectious cause of visual impairment, causing multiple pathologies such as herpes stromal keratitis, which is an immunopathological response caused by recurrent HSV infection of the cornea [184]. This viral form is the most destructive and can lead to blindness due to progressive corneal scarring with recurrences. Herpes stromal keratitis is characterized by progressive leukocytic infiltration, opacity, and vascularization of the cornea [183,185]. HSV-1 infection can be classified into primary or recurrent disease. For primary HSV-1 ocular infection, clinical manifestations tend to occur in youth or young adults. After primary infection of the oral-facial region, including the cornea, infected humans are likely to carry a latent viral load because the HSV virus moves particularly to the trigeminal ganglia, where a latent state is established without the production of infectious viral particles [57,185]. Subsequently, the virus may undergo cycles of reactivation, causing recurrent viral or immune pathology at the initial site of infection. Thus, frequent attacks of this virus cause nerve damage that reduces the sensitivity of the eye. Much work is being devoted to the HSV-1 study because a thorough understanding of the HSV-1 disease process could lead to the prevention of acute HSV-1 infection, reactivation, development of HSV-1 vaccine, and more effective treatments of recurrent eye diseases in general [57,185,186]. Clinical trials for vaccines against this type of infection have been ongoing for more than three decades [186]. Despite this, no vaccine has been approved, and no formal clinical trials have evaluated the impact of HSV vaccines on ocular health. Compared with other external anatomic sites, the pathology and healing of corneal tissues after HSV infection is complex and clinically problematic due to the need to preserve corneal clarity and sensitivity, especially in the cases of severe HSV infection, i.e., herpes stromal keratitis [185,187,188]. This disease is often studied to develop an effective vaccine against HSV-1 because if the vaccine protects against herpes stromal keratitis, it should also protect against other herpes infections in the eye. Therefore, the development of a vaccine is of practical interest against HSV and would confer immunological protection without causing irreversible corneal immunopathology, which is of paramount importance to clinicians and patients.

#### 2.2.2. Animal Models

Primary and latency HSV-1 corneal infections have been studied in a variety of animal models to better understand multiple aspects of HSV biology, molecular biology, pathogenesis, disease, and immunity, especially for vaccine-induced protection [57,185,186]. Although all animal models are inherently imperfect representations of human disease, the high species specificity of HSV-1 allows for the development of a wide range of animal models, such as mice, rabbits, guinea pigs, rats, owl monkeys, and rhesus macaques. These models exhibit many characteristics of human HSV-1 corneal disease and are dependent on important experimental parameters, including species, age, and genotype of the animal, the route of infection, as well as the viral serotype, strain, and dose [57,182,183]. The most popular animal models have been developed in mice and rabbits, followed by guinea pigs, to study ocular HSV-1 latency, reactivation, and recurrence in immune responses and pathogenesis. Biosafety level 2 laboratory facilities with adequate practices and procedures are also required to study HSV and manipulate and house animals. In the case of studies on the mechanisms of HSV infection using animal models, such as the establishment and maintenance of viral latency, these experiments very often require that the animals be kept alive for at least a month in most cases and longer in certain circumstances, resulting in a non-negligible cost [182]. For long-term experiments, a proportion of infected animals will experience significant morbidity and may progress to a fatal outcome, either by acute spread of the virus before the establishment of latency or by reactivation of the latent virus. Furthermore, an important parameter to be considered is the age of the animals because resistance to HSV disease is reduced in young animals whose immune and adaptive response is more vulnerable [182]. Some ocular tissue inoculation approaches are well suited to establish HSV disease as a peripheral infection. Viral inoculum is injected into normal or scarred corneas to facilitate viral uptake and mimic sites of primary human ocular HSV-1 infection. The disease progression is measured by examining corneal opacity and lesions [182]. Using this invasive model, core body temperature, coat appearance, weight, posture, movement capacity, and aggressiveness are measured to assess the animal’s pain and discomfort and visually distinguish moribund animals from those that appear normal. After initial replication in the periphery, HSV-1 infects the ophthalmic branch of the trigeminal nerve and can be detected in the trigeminal ganglia within 2 to 3-days of infection [189].

The following sections focus on the most studied animal models for ocular herpes, i.e., mouse and rabbit models. In both of these models, primary corneal infection with appropriate strains of HSV-1 results in epithelial damage in most animals and subsequent development of herpes stromal keratitis in a portion of them similar to what can be observed in human corneas [183,190]. Indeed, corneal ulcers can also have a punctate, dendritic, or geographic (i.e., enlargement and fusion of ulcers) appearance when stained with fluorescein, rose Bengal, or lissamine green and a stromal inflammation without an associated epithelial defect may also subsequently occur in these animal models, named immune herpes stromal keratitis.

##### Mouse Models

Several features make mouse models excellent candidates for studying HSV-1-induced immune responses during latency, reactivation, and recurrent HSV-1 infection. Although murine models of ocular primary infection are intrinsically different from human herpes stromal keratitis, many parallels can be drawn to clarify vaccine efficacy. Murine models are used in basic research to assess the ocular pathogenesis of acute HSV 1 infection and characterize immune responses [186,191]. The availability of inbred and transgenic strains for studying this pathology is greater than for other species, and reagents are also available to dissect the immune response to HSV-1 [57,182,183,185]. The knockout mouse models used for ocular herpes involved the elimination of components of the immune system and are used to study the effects of the immune system on latency and reactivation. These transgenic mouse models have provided insight into the role of specific genes and cytokines involved in HSV-1 ocular disease, i.e., HSV-1 latency, reactivation, and recurrence [57,182,183]. Among these models, Human *ApoE3^+^/^+^* and Human *ApoE4^+^/^+^* knockout mice were developed to study the role of human *ApoE4* [192]. This gene plays a role in the establishment of HSV-1 latency, being implicated in the pathogenesis of ocular herpes and the immune response of microglia. *ApoE* knockout mice are resistant to the neurovirulence of the HSV-1 strain 17Syn^+^ after corneal inoculation of the HSV-1 strain, whereas wild-type mice showed a latent load of the virus in the digestive tract. Other transgenic mouse models have been used to study ocular HSV-1 infection, such as *IL-1ra^−^/^−^* (role of IL-1 in HSV-1 stromal keratitis) [193], *p19^−^/^−^* (role of IL-23 in the severity of HSV-1 ocular lesions) [194], wild-type and *p53^−^/^−^* (role of p43 in HSV-1 replication) [195]. One of the main limitations of the mouse model, particularly for vaccine development, is that the virus does not spontaneously reactivate and is not excreted on the surface of the cornea in mice, unlike in rabbits and humans [182,183]. It has been stipulated that the reactivation process in mice is more effectively blocked, notably by CD8^+^ T-cells resident in the trigeminal ganglia and/or that mice are less sensitive to stimuli that induce reactivation. However, it is possible to induce HSV-1 reactivation from latency, shedding, and recurrent herpes stromal keratitis in mice, but the protocols involve raising the body temperature to dangerous levels or exposing the cornea to ultraviolet light [196,197]. It is difficult to detect HSV-1 infection and HSV-1 DNA because the volume of the mouse tear film is very small [57,183,184]. The spontaneous shedding rate of HSV-1 DNA in mice is extremely low, and there are no known reports of spontaneous recurrent lesions in immunocompetent mice. In addition, infection of the mouse corneal epithelium with most strains of HSV-1 requires some degree of scarring [183]. HSV-1 reproducibly establishes latency in the mouse model from the earliest stages of acute infection, with the viral genome reaching the neuronal ganglia within the first 24 h of infection [198]. Thus, mice represent an important starting point for assessing the effect of several experimental parameters on the development of HSV disease.

##### Rabbit Models

Rabbit models also have specific advantages and disadvantages for studying ocular HSV-1. Rabbit strains that can be used for ocular herpes studies include the New Zealand White, Dutch Belted, and other pigmented rabbits [2,57]. Most strains of HSV-1 can infect all the previously mentioned rabbits. In addition, the main advantage of rabbit models, which also applies to guinea pig models, lies in their ability to spontaneously produce HSV-1 reactivation from latency and ocular surface shedding, as in humans, which does not appear to occur spontaneously in mice [57,182,183,199]. Spontaneous reactivation of HSV-1 in rabbits includes viral shedding in saliva and tears, as in human [200]. Thus, the HSV-1 infection in rabbits is more representative of human disease than that of mice [2,57,201]. Latent rabbits with high phenotype reactive strains have a high rate of spontaneous HSV-1 shedding, and their lesions share similar characteristics with human HSV-1 lesions [202]. Because herpes stromal keratitis infection in humans represents recurrent herpetic disease resulting from HSV-1 reactivation from latency in the trigeminal ganglia and shedding at the cornea, the ability to induce its recurrent form is a useful feature of the rabbit model [183]. Because of this unique feature, this model has also been widely used to evaluate the efficacy of HSV vaccines in controlling the recurrent phase of the disease [186,203]. Despite the very limited availability of transgenic rabbit models, Chentoufi et al. introduced humanized HLA-A*0201 transgenic rabbit model for the purpose of developing a vaccine against primary ocular herpes by studying HSV-1 infection [202]. This transgenic rabbit model produces human HLA-specific and restricted T-cell responses for the study of vaccines based on human CD8^+^ T-cell epitopes [202]. In this study, the human herpes lipopeptide vaccine formulation contains three pairs of peptide epitopes derived from the sequence of HSV-1 glycoprotein D and protects against ocular HSV-1 infection. This humanized transgenic rabbit model produces HSV-1-specific CD8^+^ T-cells and shows a reduction in recurrent HSV-1 disease after induction of latent HSV-1 infection when it is immunized with the vaccine. Thus, humanized animal models are a welcome advancement in this field, as previous glycoprotein D subunit vaccines have shown promising results in protection against HSV-1 and/or HSV-2 [186,191]. Consequently, it is increasingly interesting and important to focus on the development of a new vaccine against HSV infection through translational research between animal models and humans and combining clinically relevant assessments of corneal pathology with immunologic studies of vaccine efficacy. Moreover, infectious epithelial keratitis generally persists longer in rabbits, which may be helpful for testing the efficacy of anti-herpetic drugs [183]. However, rabbit strains are very expensive and difficult to obtain, and the relative lack of reagents to dissect the immune response is an additional limitation of this model [57,182,183]. These issues are important drawbacks in attempting to understand an immunopathological process such as herpes stromal keratitis with a genetic contribution from the host.

### 2.3. Corneal Repair and Transplantation

#### 2.3.1. Pathology

**Figure 6:**
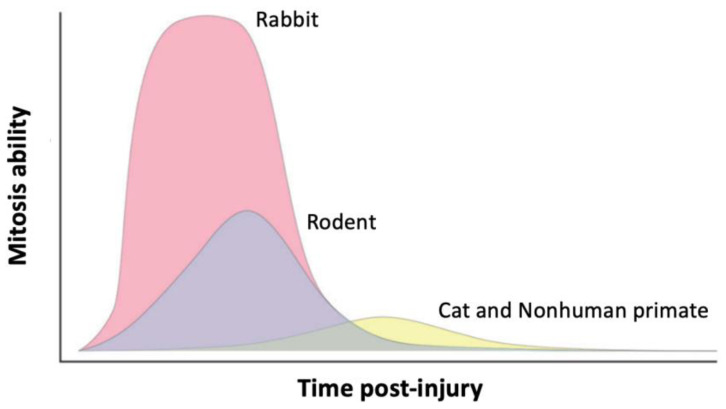
The mitotic capacity of corneal endothelial cells varies depending on the species. The regenerative ability of the human corneal endothelium is consistent with cat and nonhuman primate models. Reproduced from [209]. Copyright (2021), Annals of Translational Medicine.

**Figure 7:**
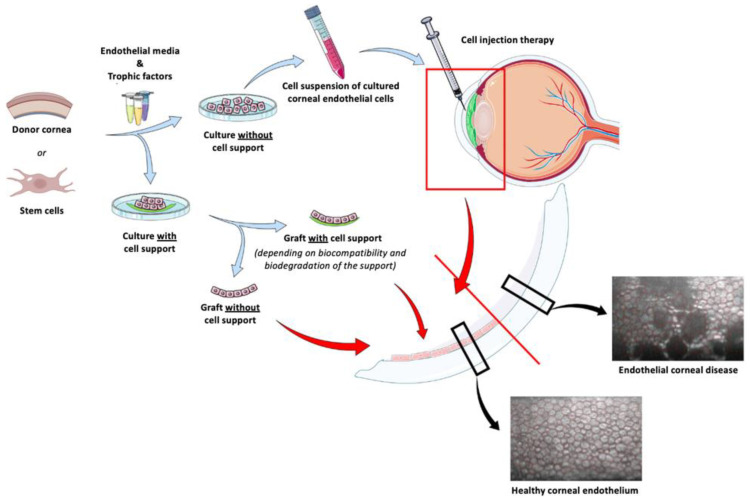
New approaches of cell-based therapy for corneal endothelial cell transplantation. Adapted from [230]. Copyright (2021), Elsevier, Experimental Eye Research.

In the United States, the eye injury rate is at least one million each year, and approximately 2000 workers undergo a work-related eye injury requiring medical treatment every day [12,204]. This is because the anatomical location of the eye makes the cornea vulnerable through continuous exposure to various abrasive forces, such as fingernails or prolonged contact lenses wear, for example, and mechanical, chemical, and thermal injuries as well as viral or bacterial infections [12,204,205,206,207]. However, overall, 75% of ocular injuries are due to foreign bodies or abrasive damages, and nearly 25% are due to chemical burns [204], which remain the most serious cause of corneal wounds [206]. In some of these corneal injuries, especially in the presence of a severe injury or when the injuries are untreated or not quickly enough, the consequences can be critical due to the limited regenerative capacity of the human corneal endothelium, requiring corneal transplantation or eye enucleation and leading to permanent visual impairment [12,204,206]. Indeed, the inability of adult human corneal endothelial cells to re-enter the cell cycle results in endothelial cell loss and decreased cell density due to injury, infection, aging, and/or disease [208,209]. Although corneal transplantation has one of the highest success rates in human transplantation, there is an urgent clinical need to improve this research field by finding an alternative to donors using animal models [12,209,210]. Data collected between 2012 and 2013 showed that only 1 in 70 patients received a corneal transplant, while more than 12 million people were waiting for a corneal transplant during the same period worldwide [12,211]. Organ donation is a complex process involving numerous social, ethical, and legal issues. As the number and techniques of corneal transplantation increase, so does the need for donor corneas, contributing to a shortage of supply and demand, particularly in developing countries. In the cornea, fibrotic repair presents unique challenges that affect both the clarity and shape of the cornea. With the increasing popularity of surgical techniques that alter corneal refractive errors, understanding the mechanisms of corneal repair has gained increasing attention [60]. The cornea has unique anatomical, cellular, molecular, and functional characteristics that result in significant mechanistic differences in the repair process compared with what occurs in the skin and other organs. When reconstructing a damaged cornea, the most important characteristics of the cornea to consider are its mechanical strength and transparency [212]. Thus, there is a growing demand for preclinical animal models of corneal endothelial dysfunction to evaluate the safety and efficacy of new therapeutics, but it depends globally on the animal model and method used to create the corneal wound (mechanical, thermal, chemical, etc.) [209]. Herein, we are mainly interested in animal models that might be most suitable for both corneal repairs based on their ability to regenerate endothelial cells (Figure 6) (i.e., the rodents [60,61,213], rabbits [12,207,209,210,214,215], cats [109,114,115,216,217], nonhuman primates [217,218,219,220] and zebrafish [208]) and for corneal transplantation as host models. In this latter case, the main applications of animal models are the evaluation of basic processes and potential treatments for transplant rejection, as well as the development of innovative approaches to transplantation as alternative solutions to eye bank human eyes, including cell-based therapies and bio-engineered corneal transplants (Figure 7).

#### 2.3.2. Animal Models

##### Animal Models for Corneal Wounds

The multi-stage process of corneal wound healing is universal for all species, regardless of the nature of the corneal injury [221], and is generally characterized by the enlargement, migration, and proliferation of cells adjacent to the wound edge [209]. However, the mitotic ability of corneal endothelial cells varies considerably between species, which impacts the rate and capacity of endothelial regeneration (Figure 6) [209]. Rabbits are most frequently used for in vivo research of corneal endothelial cell therapy since they share characteristics with the human corneal endothelium, such as diameter (which allows the use of the same surgical instruments as in humans), repair mechanisms, thickness, and composition [222], but also by their human-like eye size, relatively low cost, and ethical considerations [78,79,217]. In addition, parameters such as corneal endothelial density, central corneal thickness, and corneal diameter decrease with age in rabbits, as in humans (Figure 1 and Table 1) [222]. Nevertheless, the rabbit cornea has corneal endothelial cells that have a high capacity for in vivo regeneration in contrast to the canine, feline, or nonhuman primate models, which have limited corneal endothelial cell proliferation mechanisms like those in human corneas [207,209,223]. The consequences of this proliferation in rabbits have shown that up to 50% of the central cornea can be repaired within 10 days [224]. This type of result suggests careful attention to the analysis of negative controls to ensure that any endothelial regeneration observed is not the product of native corneal endothelial cell proliferation. The use of rabbits as a model for human corneal healing is limited due to their ability to recover from injury, making it difficult to establish the true efficacy of the treatment tested [222]. Several studies are using nonhuman primate and feline models to overcome these drawbacks [115,207,209,217]. Nevertheless, the use of older rabbits, at least 9 to 12 months of age, may be an appropriate option because they have shown a lower corneal endothelial cell density than younger rabbits [225,226]. Going further, Valdez-Garcia et al. showed that 18-month-old New Zealand White rabbits (young adults) are a suitable model for studying human corneal endothelial repair since the mitotic activity of these rabbits decreased significantly with age [222]. This model did not show mitotic activity 72 h after cryoinjury, confirming the delay in corneal endothelial healing in older rabbits. Murine models can also be used for corneal repair [227,228,229]. To enable us to take advantage of these features, Fini et al. developed a mouse model of penetrating keratectomy (surgical or laser removal of part of the cornea) by adapting a previously successful rabbit model, even though mouse eyes cause problems for surgical manipulation [60]. Another study highlighted that mouse models generated by genetic targeting and/or transgenic techniques are valuable tools to elucidate the role of proteins in the extracellular matrix for corneal wound repair [61]. Finally, an interesting study showed that the zebrafish corneal endothelium can rapidly repopulate on its own and re-enter the cell cycle, after surgical injury, to repair the wound, unlike humans. Thus, the zebrafish model has the potential to regenerate most of its corneal endothelial cells and examine whether the signaling pathways act differently in the injured zebrafish cornea. Indeed, the ease of genetic manipulation of the zebrafish allows for the study of the molecular mechanisms of corneal endothelial regeneration in vivo, which is not possible in other model systems, and understanding the mechanism of cell cycle arrest in human corneal endothelial cells [208].

##### Animal Models for Corneal Transplantation

Although humans normally have a sufficient corneal endothelial cell density for a lifetime, they may have excessive corneal endothelial cell loss because of previous eye surgery or some pathologies (e.g., Fuchs endothelial corneal dystrophy; Section 2.5), posterior polymorphic corneal dystrophy, herpetic viral infections, trauma, or elevated intraocular pressure, which can cause irreversible corneal endothelial dysfunction and decompensation, and thus loss of transparency and corneal blindness [217]. Traditionally, full-thickness corneal transplantation has been the only choice for restoring vision. Nevertheless, other corneal diseases, such as corneal neovascularization (Section 2.4), can cause graft rejection. Alternative corneal endothelial cell therapies have also attracted considerable research interest to circumvent the routine use of human donor corneas (limited number of donors, lack of quality, and complexity of surgery) or avoid the use of full-thickness corneas. Over the last decade, endothelial transplantation has thus emerged, notably to avoid graft rejection, primary graft rejection, and progressive decrease in cell density [35,207,231,232]. Appropriate models are needed to evaluate the results of cell therapy and determine the safety of the procedure to restore corneal endothelial cell function. Differentiation of pluripotent stem cells and generation of corneal endothelial cells from other cell sources as animals are very promising approaches for the development of cell therapy to treat corneal endothelial disease [35,207,230]. Endothelial cell transplantation from different human or animal cell sources and recipients as a host animal is possible because of the immune privilege of the anterior chamber, especially in the case of host-incompatible grafts that would otherwise be rejected at other transplantation sites [35,230]. Currently, the two main methods investigated to deliver live corneal endothelial cells with sufficient potential to adhere to the posterior cornea are the injection of cells into the anterior chamber of the eye and the implantation of carriers or scaffolds to perform bioengineered corneal endothelial grafts (Figure 7) [207,210,217,230]. On the one hand, the cell injection method relies on the simplicity of the technique and could be implemented worldwide, even in regions without access to highly trained corneal experts. On the other hand, the carrier materials (e.g., membranes of amniotic, silk fibroin, collagen I, gelatin, or hydrogels) must meet certain criteria such as biocompatibility, optically transparency, ease of surgical manipulation, and demonstration of mechanical properties like those of the native cornea [35,207]. Animal models offer a multi-level approach, integrating macro- and micro-environmental influences, and these models are also necessary to specifically investigate surgical transplantation or implantation of corneal endothelial cells, as well as to investigate issues including biodegradability, immune-tolerance, and long-term outcomes. The most common recipient animal species for in vivo testing are rabbits, followed by rodents, nonhuman primates, and felines, while the most common species used for in vitro corneal endothelial cell culture and transplantation are humans, followed by rabbits, nonhuman primates, felines, and murine [207,210,217,230]. Murine models have provided a better understanding of the pathogenesis of immune rejection and a wealth of information on the immune graft rejection process, thanks to the different rodent strains available [35,217,233]. In the event of rejection, the graft then becomes opacified due to an immune reaction [35,233]. This opacification is the result of immune cell-induced damage to the graft endothelium, leading to edema. A temporary corneal opacification is observed in mice and rats in the days following graft transplantation [35]. Applying clinical criteria for graft rejection, this temporary opacification may be significant enough to be considered complete graft rejection. Thus, the opacification degree and duration are necessary to determine non-reversible rejection. The C57BL/6 and BALB/c mouse strains are the most widely used to investigate the innate immune system and immunological processes during graft rejection. Recently, a study compared several aspects of corneal rejection using these mouse models [233]. The results indicated that M1 macrophages appear to play a crucial role in the rejection process. Furthermore, the authors suggest that the BALB/c recipient model could be used as a surgical control for corneal transplantation experiments, while models using C57BL/6 as recipients can serve as transplantation models in a clinical context considered “high-risk” due to severe inflammation and a high rejection rate. Strain-dependent differences then convey different innate immune responses in BALB/c and C57BL/6 strains, suggesting the mouse lineage of donor and recipient animals must be carefully considered. However, from a clinical point of view, mouse and rat models show significant anatomical differences from human grafts [207,210,217,230]. It also remains unclear to what extent these models mimic the immunological mechanisms of corneal graft rejection in humans, and whether differences in the innate immune systems of these two mouse strains affect outcome after corneal transplantation [35,233]. Consequently, the dissimilarity between murine and human immune systems, as well as the inherent size difference between the species, have made the use of larger animal models essential. Indeed, larger animal models facilitate surgery and allow the use of clinical techniques familiar to the ophthalmologist to assess graft rejection and study changes in the endothelium [35,207]. In addition, the selection of the animal model that most closely resembles human anatomy and physiology is desired to facilitate the transfer of developments from the animal model to humans when creating the corneal endothelium, especially for carrier implantation [35,207,230]. In contrast to murine models, the use of larger animal models, such as rabbits, is primarily intended for the study of graft rejection, enabling corneal transplants to be performed that will be more easily transferable to human transplantation due to their similar eye size between large species (Figure 1 and Table 1). The rabbit shows various signs of rejection also seen in humans, such as retro-corneal membranes, epithelial decompensation, and neovascularization of the graft [35]. It has also been shown in rabbits that graft size and location contributed to rejection. Indeed, widespread rejection was observed for 7-mm grafts [234], whereas smaller grafts, 4 to 5 mm, did not appear to induce rejection since the grafts retained their transparency [235]. Also, grafts placed closer to the limbus have higher rejection rates, particularly in the case of vascularization [235]. Finally, although the ideal research model for human application is the nonhuman primate for obvious reasons, in 2016, Bostan et al. showed in vivo functionality of a corneal endothelium transplanted by cell injection therapy in a feline model as an intermediary model and were able to restore corneal clarity and thickness up to 7 days post-transplant [115]. To date, the different animal models have proven to be complementary in providing researchers and clinicians with a means to develop new surgical techniques as well as to evaluate the function of various corneal grafts and new cell therapies.

### 2.4. Corneal Neovascularization

#### 2.4.1. Pathology

The avascular structure of the cornea (no lymphatic or blood vessels) is one of the main reasons for the optical clarity of the cornea and appears to be an “angiogenic privilege” against corneal infiltration by blood vessels [236,237]. Homeostasis exists in the cornea, where pro-angiogenic and anti-angiogenic factors are in equilibrium. Up-regulation of proangiogenic factors accompanied by down-regulation of antiangiogenic factors prompts the formation of new blood vessels in the avascular corneal stroma, called corneal neovascularization. Thus, hypoxia or inflammation (secondary to infection, trauma, or graft rejection) triggers the production of growth factors and angiogenic signals in response to tissue aggression [236,238,239,240]. These new vessels, initially immature and poorly structured, diffract light and introduce proteins, lipids, and inflammatory cells that disrupt corneal immune privilege, promote further inflammation, prompt graft rejection and corneal scarring.

#### 2.4.2. Animal Models

Overall, the main models for corneal neovascularization are from rabbits, rats, and mice for this type of pathology [1,238,240,241,242]. Many different methods have been used to induce corneal neovascularization in animals. Among them, alkali burn and suture placement are the two most widely used models for studying mechanisms and developing therapies [240,243,244,245,246]. In the alkali-induced model, corneal neovascularization can be induced by placing NaOH-soaked paper on the ocular surface of the animal for 10 s. In the suture-induced model, corneal neovascularization can be triggered by suturing two 10-0 nylon stitches directly onto the cornea [245]. In both models, corneal neovascularization appears and progressively extends over the first two weeks following induction of neovascularization in the animal, with up-regulation of vascular endothelial growth factors (VEGF). A strategy for treating corneal neovascularization is to inhibit VEGF activity by competitively binding it to an anti-VEGF antibody [240,244,245,246]. VEGF is one of the main factors involved in the pathogenesis of corneal neovascularization. The use of animal models is, therefore, essential to establish safe doses and administration methods before these agents can be used in the clinical context and justify further development of these agents. The efficacy of anti-VEGF agents depends on how quickly treatment is initiated after the onset of corneal neovascularization. Early administration of treatment on day 1 post injury inhibits corneal neovascularization more effectively in an experimental rabbit model of limbal insufficiency than when the treatment is administered on day 14 post injury [247]. However, treatment of corneal neovascularization with the anti-VEGF antibody has certain limitations. It is only a symptomatic treatment for corneal neovascularization and does not cure the cause of the disorder. In some cases, repeated treatment is required to maintain the drug’s positive effect over a period of time [244]. In addition, the affinity of anti-VEGF agents for VEGF needs to be considered in animal models since it may be lower than in humans, as is the case in rat models [244]. Consequently, further research in animal models is required before anti-VEGF agents can become key therapeutic agents in the inhibition of corneal angiogenesis. Some studies have shown that the use of gold or silver nanoparticles can improve therapeutic treatments of corneal neovascularization in murine models by increasing drug residence time and targeting [248,249,250]. In a study, these nanoparticles functionalized with a heparin derivative demonstrated efficacy as anti-angiogenesis agents [248]. Moreover, the use of gold nanoparticles, topically applied, significantly reduced the development of corneal neovascularization induced by alkali burn, without any significant side effects, by inhibiting the extracellular signal-regulated kinase pathway [249].

### 2.5. Corneal Dystrophy

#### 2.5.1. Pathology

Corneal dystrophies represent a heterogeneous group of genetic diseases generally describing rare inherited disorders of the cornea that are bilateral and often symmetrical, slowly progressive, and unrelated to environmental or systemic factors [251,252,253,254]. They are characterized by abnormal accumulations of insoluble deposits in different layers of the cornea and affect cells, tissues, and/or organs. However, there are many exceptions, as not all corneal dystrophies meet these criteria [251]. In 2015, the International Committee for the Classification of Corneal Dystrophies (IC3D) revised the anatomic classification of corneal dystrophies (epithelial and subepithelial, epithelial-stromal TGFBI, stromal, and endothelial), and this classification also identifies corneal dystrophies into four categories based on clinical, pathologic, and genetic information [251,252,253]. Moreover, many dystrophies involve more than one corneal layer. A total of 22 distinct forms of corneal dystrophies that are inherited through autosomal dominant patterns can be distinguished, although autosomal recessive and X-chromosomal dominant patterns also exist [251,254]. The symptoms of patients with corneal dystrophy are highly variable [252]. Many of them do not show any symptoms, especially at the beginning of the disease. Patients usually have recurrent epithelial erosions resulting in morning eye pain and discomfort for those in whom the more superficial layers are affected [251,252,254]. In contrast, patients with stromal dystrophies tend to have reduced visual acuity due to deposits of abnormal substances in the main area of the cornea. Vision loss is also the primary symptom in patients with endothelial corneal dystrophies due to fluid retention (swelling of the cornea), leading to corneal edema which results in progressive loss of corneal transparency [252,254,255,256,257,258]. This type of corneal dystrophy accounts for approximately 60% of all types of corneal dystrophies [252]. The discovery of the genetic basis of corneal dystrophies is not complete, and the molecular mechanisms of the different mutations in the pathogenesis of each corneal dystrophy remain unclear. The development of gene therapy in the initial stages of corneal dystrophies is an important scientific challenge for the future. In contrast to retinal dystrophies, corneal dystrophies are more amenable to such therapy because of the anatomical accessibility of the cornea [254].

Fuchs endothelial corneal dystrophy (FECD) is the most common corneal dystrophy, with a prevalence ranging from 3 to 11% depending on the age, ethnicity, and sex of the population [255,256,257,258,259]. This genetically heterogeneous disease is the most frequent cause of corneal transplantation worldwide. Two forms of FECD exist—the rare early-onset form and the more common late-onset form [252,255,257]. Although the primary cause of this disease is unknown [254,260], this bilateral disease of the corneal endothelium is characterized by accelerated loss of corneal endothelial cells and the formation of extracellular matrix excrescences in Descemet’s membrane, called guttae [256,257,258,259]. Endothelial cell oxidative stress, apoptosis, loss of pump function, and deposition of abnormal extracellular matrix occur in the initial stages of the disease. These responses are manifested by endothelial cell loss, enlargement, and change in morphology associated with Descemet’s membrane thickening and guttae formation, leading to corneal edema until vision is lost. Although FECD is primarily a disease of the corneal endothelium, secondary changes may eventually affect all layers of the cornea, such as the stromal and epithelial layers, as well as the corneal nerves [255,258]. It is inherited in an autosomal dominant mode, with incomplete penetrance and a female predominance [255,257,258]. Other corneal diseases described in this review could be associated with FECD, such as diabetes mellitus (DBMT; Section 2.6) and keratoconus (Section 2.7) [251,257].

#### 2.5.2. Animal Models for Fuchs Endothelial Corneal Dystrophy

Currently, there is no treatment for FECD other than corneal transplantation (Section 2.3) [259]. Descemet’s membrane stripping is a major step in endothelial keratoplasty techniques for this disease in the context of corneal transplantation. Preclinical animal models of Descemet’s membrane stripping have been used to evaluate various biological and synthetic support materials seeded with cultured corneal endothelial cells [209]. A cellular approach has been used to replace corneal endothelium and involves delivering cultured human corneal endothelial cells as a cell suspension directly into the anterior chamber by intracameral injection after removing native corneal endothelial cells by stripping the Descemet’s membrane [255,257,260,261]. Okumura’s group demonstrated that Descemet’s membrane stripping, in combination with cultured corneal endothelial cells injection, is feasible for FECD patients to further improve visual quality using a rabbit and monkey model [220,262]. The application of a Rho-kinase (ROCK) inhibitor Y-27632, after injection of cultured corneal endothelial cells in monkey and rabbit models, has been shown to improve cell adhesion and proliferation by regenerating healthy corneal endothelium [262,263]. This method also restored vision and maintained corneal transparency without any drug side effects, such as persistent epithelial defects or corneal stromal scarring. Researchers have achieved similar results by implanting human corneal endothelial cells in primates [220]. Y-27632 also effectively reduces FECD-induced central corneal edema in a small group of patients [264] and improves wound healing in rabbits and primates [264]. A study also showed that after culturing normal corneal endothelial cells and FECD cells to create corneal endothelium, the modified FECD corneas were transplanted onto devitalized human stromal media into feline eyes and were able to restore corneal clarity, observed with a slit-lamp biomicroscope, and corneal thickness up to 7 days post-transplant in these animals [115]. These tissue engineering models demonstrate that cell therapy with human corneal endothelial cells delivered as a cell suspension to the anterior chamber can produce in vivo functional corneal endothelium and is well tolerated [257]. They also make it possible to study the behavior of FECD cells in a healthy environment as well as to analyze and understand the initial events of this disease. In addition, injected corneal endothelial cells in the context of corneal transplantation represent an important source of FECD cells to study the late cellular events of the disease [258].

Animal models allow for the study of the genetics of FECD at different stages of the disease and/or in the presence of external stress factors, as well as for the testing of new treatments for FECD [209,256,257,258,260]. The genetic basis of FECD includes many genes and chromosomal loci, although alterations in the *TCF4* gene are responsible for approximately 70% of FECD cases [209,255]. Although mutations causing early-onset FECD have been exclusively linked to the α2 chain of collagen VIII (COL8A2), which is a major component of Descemet’s membrane, the *TCF4*, *TCF8*, *SLC4A11*, *ZEB1*, *LOXHD1* genes have been implicated in late-onset FECD [209,261]. A homozygous double knockout mouse model for *COL8A1* and *COL8A2* was generated [265]. The authors observed anterior segment dysgenesis of the eye and anterior chamber protrusion was observed, as well as thinning of the corneal stroma and Descemet’s membrane. However, no guttae were observed, and there was no evidence of corneal opacification. Homozygous knock-in mouse models containing a point mutation homologous to the human Q455K and L450W mutation in the *COL8A2* gene were also generated as these missense mutations have been shown to cause early-onset FECD in humans [266,267]. These knock-in mice showed endothelial phenotypes like human FECD at early onset, including altered corneal endothelial cell morphology, their loss, guttae formation, endoplasmic reticulum stress, and activation of the corneal endothelial unfolded protein response [209,257,258]. Induction of autophagy by lithium administration and *N*-acetylcysteine ingestion via drinking water reduce both endoplasmic reticulum and oxidative stress and increase corneal endothelial cell survival in Q455K and L450W mouse models [268,269]. Taken together, these results support a pathogenic mechanism in which early-onset FECD is the result of endoplasmic reticulum stress and unfolded protein response-associated apoptosis rather than a loss of function of *COL8A2*. To investigate the role of various genes (*TCF4*, *SLC4A1*, and *ZEB1*) involved in late-onset FECD, genetically engineered mice were also generated [209,257]. Mouse models have been used to study the *TCF4* gene [257]. Although the *TCF4* heterozygous mouse models are viable, *TCF4* homozygous knockout (*TCF4^−^/^−^*) mice die within 24 h of birth, indicating that *TCF4* is a crucial transcription factor required for normal development [270]. In addition, *TCF4^−^/^−^* mice do not have any anatomical defects, including no specific ocular abnormalities, which limits the ability to study corneal endothelium [271]. However, *TCF4^−^/^−^* mice used to study FECD showed disturbed hindbrain development that is not characteristic of FECD [257]. In addition, researchers have focused on the *SLC4A11* gene that encodes NaBC1 [257,272]. Mutations in the *SLC4A11* gene have been shown to cause either congenital hereditary endothelial dystrophy or combined hearing and vision impairment [272]. Therefore, a homozygous mutant *SLC4A11* knockout mouse model [273] was developed to have a mild corneal phenotype, with no significant difference in corneal endothelial morphology and without any opacification or edema but significant abnormalities of the audio-vestibular system. However, the main phenotypic change observed in the cornea was an increase in the absolute and relative height of the basal corneal epithelial cells [273]. The lack of a severe phenotype in the cornea could be due to compensatory mechanisms in the knockout mouse or because NaBC1 does not play a direct role in maintaining corneal clarity in the mouse, despite its strong correlation with FECD and congenital hereditary endothelial dystrophy in humans. Nevertheless, understanding why mouse corneas do not exhibit a dystrophic phenotype may provide insight into the molecular basis of FECD, as well as conditions such as congenital hereditary endothelial dystrophy. Homozygous and heterozygous *ZEB1* knock-out mutant mice were generated that exhibit ectopic expression of epithelial genes in the corneal endothelium and keratocytes. These mice also exhibit more features of posterior polymorphic corneal dystrophy, rather than that seen in FECD [274]. Therefore, the development of effective genetic interventions to treat corneal dystrophies is hampered by the lack of animal models resembling human corneal dystrophies, making it difficult to evaluate in vivo treatments.

Another approach is being considered to develop new animal models of FECD. Corneal endothelial cells are particularly sensitive to oxidative stress, which is the case in FECD patients due to their chronic exposure to UV radiation and the high oxygen demand associated with active pump function [209]. In general, the severity of UV-induced tissue damage depends on the wavelength and intensity of the light and the absorption spectrum of each tissue. In addition, UV irradiation leads to a slow onset of corneal endothelial cell damage [209]. The effect of UVA has been shown to be an important etiologic factor in FECD pathogenesis, explaining the predominantly central location of cell loss and guttae formation in FECD patients [257,275]. Gene expression studies of corneal endothelial cells from human FECD patients and an FECD mouse model provide evidence of accelerated senescence as a potential consequence of oxidative stress [258]. To mimic a pro-oxidative environment leading to DNA damage and resulting corneal endothelial dysfunction, UV irradiation was applied to animal corneas [209,257,258]. At the molecular level, UVA exposure induced delayed nuclear DNA damage with low corneal endothelial cell density in mice starting only one month after irradiation [257]. UVB irradiation induced alterations in corneal endothelial cells in mice, rats, and rabbits, with corneal endothelial cells apoptosis and corneal edema [209]. Liu et al. recently developed a nongenetic UVA-induced FECD mouse model [275]. The corneal UVA exposure time was adjusted to obtain the appropriate fluence, i.e., 250 J/cm^2^, 500 J/cm^2^, 750 J/cm^2^, and 1000 J/cm^2^. To simulate the life-long exposure of endothelium to UV light, the authors used high-dose UVA irradiation (1000 J/cm^2^) and detected progressive degenerative effects of UVA-mediated damage. This in vivo model characterizes FECD in patients as morphologic changes, corneal endothelial cells loss, Descemet’s membrane thickening, and guttae-like lesion formation [209,257,258,275]. This late-onset mouse model, based on the corneal endothelial cell sensitivity to oxidative stress, simulates the female predisposition as observed in FECD patients and showed more pronounced cell loss and corneal edema in female mice at a lower dose of UVA compared with male mice [275]. This method may be the most physiologically relevant inducible animal model of FECD currently under development and provides a tool to study potential therapeutic interventions for all forms of FECD, regardless of genotype. In the same idea, the study of corneal endothelial dystrophy in a canine model suggests the underlying presence of an inherited component by sharing clinical and histologic similarities with corneal endothelial dystrophy in human patients [209]. However, it is important to note that preclinical studies in laboratory animals are often poor predictors of human clinical trials. This is due to the strictly regulated breeding environments, their highly inbred and uniform genetic backgrounds, and the lack of accounting for environmental factors associated with the risk of developing corneal endothelial dystrophy, such as smoking, diabetes, and cardiovascular disease. To improve the relevance of these animal models, it is crucial to consider and expose them to the same epigenetic factors as their human counterparts [209].

### 2.6. Diabetic Keratopathy

#### 2.6.1. Pathology

Type 1 (insulin-dependent) and type 2 (non-insulin-dependent) diabetes mellitus (DBMT) is a chronic metabolic disorder characterized by increased blood glucose levels due to insulin deficiency [276]. Currently, approximately 415 million adults worldwide are diagnosed with DBMT, and over 640 million people are expected to develop DBMT by 2040 [277]. Although retinal and lens abnormalities have been extensively studied in eye research, corneal disorders secondary to DBMT, i.e., diabetic keratopathy, are increasingly recognized as a cause of DBMT-related morbidity [277,278,279]. Diabetic keratopathy, which is often underdiagnosed, appears to be more common and may affect up to 70% of diabetic patients examined during their disease [278,279]. Diabetic patients may have corneal alterations and an increased risk of developing corneal epithelial fragility, such as tear film changes, corneal epithelial abnormalities, neurotrophic keratopathy, recurrent epithelial erosions, loss or decrease of corneal sensitivity, abnormal epithelial healing, increased susceptibility to wounds, increased postoperative surgical complications (e.g., cataract) and unhealed or infected corneal ulceration [277,278,279,280]. Consequently, these healing difficulties expose patients to ocular complications such as surface irregularities, corneal infections, and stromal opacification, which can lead to visual impairment. Various treatments can be performed for the patient with diabetic keratopathy to minimize the impact of the disease, such as increasing the lubrication of the corneal surface, preventing infection of the corneal epithelial defects by prophylactic antibiotic eye drops, or reducing exposure to prevent corneal melting [279].

#### 2.6.2. Animal Models

##### Induction of Diabetes in Animal Models

Animal models of type 1 and type 2 DBMT have been well established in rats, mice, rabbits, monkeys, dogs, cats, and pigs, all of which mimic the relatively early signs of human diabetic eye disease and provide a better characterization of diabetic corneal complications [278,281]. The diversity of current diabetic models allows DBMT to be presented with more than one model, which corresponds to the diversity of clinical manifestations in diabetic patients. Different methods have been thus developed to induce DBMT in diabetic animal models, such as surgical removal of the pancreas, streptozotocin, and alloxan administration, genetic manipulation, high galactose diets, and laser- or chemical-induced eye damage [8,9,282,283,284]. On the one hand, induction of diabetes by alloxan is considered less effective, and models induced by genetic manipulation, surgery, and lasers are the most difficult to implement. On the other hand, streptozotocin administration is the most widely used method, as it leads to the most rapid development of the disease, and dietary methods are the ones that require the longest time for the disease to progress (several years). Nevertheless, the major drawback of current studies in preclinical trials of new drugs is the predominant use of type 1 (insulin-dependent) diabetic animal models, whereas most diabetic humans are type 2 (non-insulin-dependent) [278]. In addition, some features of diabetes in the animal cornea have been shown to differ from the human disease [278]. As an example, diabetic rabbits show degradative changes in the basement membrane, as in human corneas, whereas diabetic rats and mice show increased staining of the basement membrane for laminin [285,286]. On the contrary, delayed epithelial healing has also been observed in rats but not in rabbits. Overall, animal models of diabetic keratopathy should be well characterized for a particular diabetic symptom or symptom group to be studied, and the data should be compared with the human situation. Many studies using animal models to treat diabetic keratopathy use the rat model, followed closely by the mouse model [8,277,278,279,280,281,286,287]. In the last decade, new factors involved in the delayed healing of the corneal epithelium in diabetes have been identified by studying the molecular mechanisms [281]. Most work has involved either intraperitoneal injection of streptozotocin in rats and mice modeling type 1 diabetes or feeding a high-fat diet to mice, causing diet-induced obesity and increased insulin resistance, modeling type 2 diabetes [281]. Two protocols were used to produce streptozotocin-induced diabetic rodents, involving multiple administrations of low-dose drugs or a single administration of high-dose drugs. Drug doses may vary according to the animal species and route of administration [288]. In the high-dose streptozotocin technique, a single injection of the drug is administered intravenously or intraperitoneally to mice (100-200 mg/kg) or rats (35–65 mg/kg), resulting in massive destruction of pancreatic β-cells with low or no insulin production [8,282,283,284]. Although various low-dose streptozotocin administration methods suggest that low doses (20–40 mg/kg/day) need to be spread over time to progress insulitis, a study showed that a rat model (male Sprague-Dawley) of diabetic keratopathy was obtained using a single dose of streptozotocin (50 mg/kg) injected intraperitoneally [8]. This model could be used to study corneal morphology, metabolism, and function in cases where it is difficult to obtain human samples. The authors monitored plasma glucose concentrations to establish the diabetic model until a glucose threshold of 2.5 g/L was reached. If this value was not reached within 3 days of streptozotocin administration, the rats received a second dose. It was also shown that corneal sensitivity decreased from the second month, and epithelial defects were more prominent in the diabetic group. In addition, studies on streptozotocin-induced diabetic rats showed a reduction in melatonin synthesis, which, therefore, seems to be a suitable model to perform preclinical studies with melatonin derivatives for the development of anti-diabetic therapies [289]. Genetic models of diabetes have also been introduced, such as the non-obese diabetic mouse, the KK mouse, and the Goto–Kakizaki rat [282,283,284]. Among these models, the non-obese diabetic mouse is one of the major genetic models of type 1 diabetes because it has several genetic and immunological characteristics like those of this human metabolic disease. These genetically modified mice develop insulitis at around 3 to 4 weeks of age and around 90% of pancreatic insulin is lost, leading to the onset of diabetes and rapid weight loss from 10th to 14th weeks [290]. Regarding obesity-associated diabetes, the genetically modified KK mouse model is widely investigated as a hereditarily fat mouse model [284]. This model has the capacity to develop type 2 diabetes in response to a high-fat diet and age, by exhibiting glucose intolerance and insulin resistance. The Goto–Kakizaki rat model is based on the selective breeding, over many generations, of non-diabetic Wistar rats with glucose tolerance [282,283,284]. Goto-Kakizaki rats are insulin-resistant, non-obese and immediately develop type 2 diabetes. Disease progression in this rat is associated with chronic inflammation and is therefore used in pathophysiology and therapeutic studies of type 2 diabetes. These rat models also reveal corneal phenotypes like those of patients with diabetes, including reduced corneal sensitivity, delayed epithelial healing, and reduced lacrimal secretion.

##### Animal Models for the Treatment of Diabetic Keratopathy

New systemic drugs have been investigated to treat diabetes [287], such as glutazumab in mice, rats, and monkeys [291]. Other drugs have also been tested as resolving-D1 to reduce corneal nerve degeneration in diabetic rats [292], β-carotene to improve diabetes-related corneal ultrastructural changes in diabetic rats [293], or a combination of α-lipoic acid, menhaden oil, and enalapril to reverse diabetic corneal and peripheral neuropathy in streptozotocin-induced diabetic rats [294]. In addition, other treatments exist, such as intensive systemic insulin treatment that establishes normoglycemia in diabetic rats and prevents the delayed healing of the ocular surface epithelium observed in poorly controlled diabetic animals [280]. It has also been reported that topical insulin treatment significantly accelerates wound healing in diabetic rats, although this method did not affect corneal wound re-epithelialization in healthy rats [280]. Therefore, the mechanism after topical insulin administration is still unclear with respect to accelerated corneal epithelialization in people with diabetes. Reports have also shown that topical or systemic application of naltrexone markedly accelerates epithelial DNA synthesis and corneal re-epithelialization in rats [295], rabbits [296], and humans [297] by blocking the interaction of the opioid growth factor with its receptor, therefore increasing cell proliferation [298]. In a streptozotocin-induced diabetic rat model, topical administration of naltrexone 4 times a day for 7 days from the 8th week after the onset of diabetes was shown to be effective over a wide range of administered drug concentrations (between 10^−4^ and 10^−6^ M) [298]. Topical application of naltrexone resulted in corneal epithelial wound healing in uncontrolled type 1 diabetic rodents. However, simultaneous application of topical naltrexone and topical insulin does not have an additive or synergistic effect on corneal re-epithelialization [280]. Another study showed that naltrexone was effective in normalizing tear production and restoring corneal sensitivity in diabetic rats [299]. Naltrexone has also been shown to successfully accelerate corneal wound healing by systemic or topical administration in normal and diabetic rats [280]. Although most of the studies cited in this review to treat diabetic keratopathy use topical ocular administration of a drug solution due to the simplicity of preparation and administration, the active drug substance is rapidly diluted by the tear film, and a large proportion is eliminated by various physical and physiological factors and could benefit from a mucoadhesive drug delivery system [300,301]. Despite the differences between the human and animal characteristics of diabetic keratopathy, the use of animal models has contributed to a better understanding of this disease and the study of more effective treatments. The close approximation between the available in vivo models and human corneas, allows the rapid transposition of the results into clinical trials.

### 2.7. Keratoconus

#### 2.7.1. Pathology

Keratoconus is a degenerative corneal pathology that usually affects both eyes. It is a complex multifactorial disease affecting approximately 1 in 2000 people worldwide and is characterized by the weakening of the cornea due to structural and/or compositional abnormalities [302,303,304,305]. Indeed, the cornea often deforms bilaterally and asymmetrically, thins, scars, and loses its spherical shape for a conical shape, resulting in distorted vision that is difficult to correct with glasses. This is especially the case for advanced stages of the disease, which often require surgery, such as corneal transplantation because the cornea is no longer able to maintain its normal shape in the face of intraocular pressure in the eye and swells outward [302,305]. Therefore, changes in structure and composition are often manifested by a change in shape as well as mechanical and optical changes. Keratoconus is one of the leading causes of keratoplasty [302]. It is easy to diagnose patients with end-stage keratoconus for corneal transplantation, but it is more difficult to diagnose early stages [303]. Currently, corneal transplantation is expensive, and sources of material are scarce. Therefore, there remains an urgent need to study keratoconus pathogenesis. Although genetic etiology is increasingly demonstrated for corneal dystrophy, the pathogenesis of keratoconus remains misunderstood and is often considered a heterogeneous disease that may involve genetic and environmental factors as well as other exogenous degenerative factors [303,304,305,306,307]. However, a growing body of evidence demonstrates the role of oxidative damage in the pathogenesis of this corneal disorder [305].

#### 2.7.2. Animal Models

The literature highlights the importance of in vitro human studies for a disease that currently lacks robust biomarkers and animal models. Only a few reports of animals with keratoconus have been published, but none of them report a transgenic animal model developed with the keratoconus phenotype, except for a case report of keratoconus in a rhesus monkey in 1987, although this study did not present any information on the pattern of inheritance [308]. For the reasons described earlier in this review, the development of a mouse model could be a relevant and viable model for understanding the genetic processes responsible for this pathology. Keratoconus-like corneas were identified in a mouse after repeated inbreeding [309]. A stable strain was established with an autosomal recessive inheritance pattern that showed androgen-dependent expression. However, the same authors also described keratoconus-like corneas in another mouse strain that, unlike the first strain, was androgen-independent [310], highlighting the difficulty of establishing animal models for keratoconus. The relatively small number of reports in the literature may be the result of the slow progression of the disease and the relatively short life span of the experimental animals, as specified by the authors of these studies [309,310], which could have resulted in keratoconus. Recently, Liu et al. investigated the role of oxidative stress in keratocytes in the pathogenesis of keratoconus using a rabbit model [305]. Briefly, female New Zealand White rabbits between 3.0 and 3.5 kg were used. A collagenase type II solution was transferred into the corneal trephines for 30 min at room temperature after epithelial debridement, while the control group’s solution was deficient in collagenase type II. Before surgery, the rabbits’ eyes were examined by a slit lamp every day for the 14-day study. Oxidative stress and compensatory activation of antioxidant proteins suggest that oxidative stress injury in corneal stromal cells plays a key role in the development of keratoconus in this rabbit model. The results of this study also suggest a therapeutic target that may provide innovative ideas for the drug treatment of keratoconus. Consequently, the key molecular mechanisms responsible for the onset and progression of this disease have not yet been elucidated, resulting in a significant need to develop models for a complete understanding at the cellular level.

### 2.8. Development of Therapeutic Devices Requiring Animal Models

Contact lenses are optical devices regulated by the Food and Drug Administration (FDA). Primary applications of contact lenses include vision correction, therapeutics, and cosmetics [311]. In these applications, the end user of contact lenses must meet requirements such as wearing time, comfort, durability, ease of handling, stability of vision, etc. [312]. The global contact lens market continues to grow, with an estimated USD 7.1 billion in 2015 [312], and could reach USD 19 billion by 2024 [311]. Indeed, to correct refractive errors in myopia, hyperopia, and astigmatism cases, there are approximately 140 million people worldwide who use contact lenses. In addition, therapeutic contact lenses are used to treat eye dysfunctions, including corneal irregularities, and for rehabilitation after refractive surgery. They can also be used as smart delivery systems to extend drug release times and as wearable biosensing platforms [311]. However, contact lens wear has some disadvantages, which can induce adverse effects such as discomfort (most common), microbial keratitis, allergies, and corneal complications (limbal epithelial stem cells deficiency, tear film stability, corneal abscess, etc.) [1,311,312]. The animal model used to test contact lenses is mainly the rabbit because its eye size is like that of humans [311]. In addition, there are international standards for in vivo biocompatibility testing on rabbit eyes, approved by the International Organization for Standardization (ISO 9394:2012; last reviewed and confirmed in 2023) suggesting inserting the lens into one eye and using the other eye as a control [313]. The irritation and sensitization properties of lens materials in contact with ocular tissue are specifically evaluated according to ISO 10993-10:2021 (revised in 2021) [314]. New Zealand White rabbits (male, female, or mixed sexes) that are young adults, or albino rabbits, from a single strain, weighing more than 2.5 kg and free of clinically significant ocular irritation or corneal retention after fluorescein staining, are preferably used [311]. The lens is left in the animal’s eye for 7 h and then removed. This procedure is repeated for 21 days to best mimic human contact lens use. Presently, it is important to pay attention to emerging therapies using contact lenses as a diagnostic tool and ocular drug delivery system. This represents an additional challenge for the use of animal models in the context of medical device regulations.

## 3. Conclusions

Animals are indispensable models for translating fundamental mechanistic findings and using their therapeutic potential in humans. Small animal models are useful for understanding the mechanisms of molecular biology and disease pathogenesis, particularly due to the wide availability of these small mutant, transgenic, and genetically modified animals, as well as their short generation time. However, the application of these discoveries to humans often requires the use of large species, more representative of the human condition, whose conditions are either experimentally induced or natural. The use of large animal models is not without controversy, including ethical issues and higher overall cost, and experiments must be carefully planned after a thorough review of the literature and consideration of alternatives. Animal models have been used for experiments since the fifth century BC [315], and the total replacement of animal testing will not happen anytime soon [316]. Indeed, even if the US Food and Drug Administration aims for a future without lab animals and authorizes the clearing of new drugs without mandatory animal testing [317], the replacement models, such as computer modeling, organ chips, and other non-animal methods, are not fully ready for all drugs and diseases. Discussions between researchers, companies, and agencies about the use of these substitutes will now increase, and, for example, software that can run thousands of compounds in an hour will be seriously considered. In this review, we have described the main experimental model species used for eye research with emphasis on the most common corneal pathologies. To date, the different animal models have proven to be complementary in providing researchers and clinicians with a means to develop new surgical techniques, therapies, etc., although the choice of animal model is guided by the experimental aims and ethical considerations, which often dictate the use of smaller animals. This review summarizes the various animal models most commonly used for studying, understanding, and benefiting patients through translational research in corneal diseases. Because preclinical studies play a key role in the decision to proceed to human clinical trials, researchers must carefully select appropriate animal models, optimize, and standardize experimental protocols to facilitate the transfer of research findings from animals to humans.

## Figures and Tables

**Table 2:** Relative comparison of animal models for corneal eye research. The description of each criterion was carried out arbitrarily based on the information gathered in the literature and reported in this review (●●●●● means that the animal model contributes more significantly to the criterion rather than ●○○○○○). DED: dry eye disease; DK: diabetic keratopathy; FECD: Fuchs endothelial corneal dystrophy; HSV: herpes simplex virus; KC: keratoconus.

Animal Models	Rodents	Rabbits	Nonhuman Primates	Pigs	Felines	Canines	Zebrafish
Average cost per animal	●●○○○	●●●○○	●●●●●	●●●●○	●●●●○	●●●●○	●○○○○
Space required	●●○○○	●●●○○	●●●●●	●●●●○	●●●●○	●●●●○	●○○○○
Breeding rate	●●●●○	●●●○○	●○○○○	●●○○○	●●○○○	●●○○○	●●●●●
Feasibility of high-throughput screening	●●●●○	●●○○○	●○○○○	●○○○○	●○○○○	●●○○○	●●●●●
Availability of mutant, transgenic, and genetically modified strains	●●●●●	●●●○○	●○○○○	●●○○○	●●○○○	●●○○○	●●●●○
Eye anatomical similarity to humans	●●○○○	●●●○○	●●●●●	●●●●○	●●●●○	●●●●○	●○○○○
Eye genetic similarity to humans	●●○○○	●●●○○	●●●●●	●●●●○	●●●●○	●●●●○	●●○○○
Study of associated corneal pathologies	DED HSV Repair Transplantation Neovascularization FECD DK KC	DED HSV Repair Transplantation Neovascularization FECD DK KC Contact lenses	HSV Repair Transplantation FECD Contact lenses	Repair Transplantation DK	Repair Transplantation FECD DK	DED DK	Repair Corneal dystrophy Drug-related oculotoxicity
